# Medical irregular multivariate time series forecasting based on multi-scale temporal-frequency domain patch fusion and dynamic graph

**DOI:** 10.3389/fphys.2026.1767037

**Published:** 2026-04-15

**Authors:** Xueping Liu, Tianyi Gong, Youru Li, Na Li, Silu Ding

**Affiliations:** 1 College of Artificial Intelligence, Shenyang Aerospace University, Shenyang, China; 2 College of Computer Science, Beijing University of Technology, Beijing, China; 3 Department of Medical Imaging, Liaoning Cancer Institute and Hospital, Shenyang, China; 4 Department of Radiation Oncology, The First Hospital of China Medical University, Shenyang, China

**Keywords:** dynamic graph, irregular multivariate time series, medical forecasting, multi-scale patching, temporal-frequency fusion

## Abstract

**Introduction:**

Accurate forecasting of medical irregular multivariate time series is an important prerequisite for downstream monitoring and decision-support research. However, this task remains challenging because physiological data are typically characterized by irregular sampling, missing values, and complex temporal and inter-variable dependencies.

**Methods:**

To address these challenges, we propose a novel method termed Multi-scale Temporal-Frequency domain fusion Patching and Dynamic Graph modeling (MTFP-DG). The method first transforms irregular time series into multi-scale patches with unified temporal resolution, enabling temporal alignment without interpolation and thereby handling irregularity and asynchrony. It then employs a dual-domain encoding mechanism that fuses temporal features extracted by a Transformable Time-aware Convolution Network with frequency features extracted by an Irregular Fourier Analysis Network to obtain rich patch representations. Based on these representations and Fourier coefficients, dynamic graphs are further constructed to capture evolving inter-variable correlations.

**Results:**

Extensive experiments on five real-world medical datasets demonstrate that MTFP-DG outperforms state-of-the-art baselines on retrospective irregular multivariate time series forecasting benchmarks.

**Discussion:**

These findings indicate that integrating multi-scale patching with dynamic graph modeling is effective for capturing complex temporal dependencies and inter-series relationships in medical irregular multivariate time series. MTFP-DG may provide a robust methodological tool for proactive healthcare planning, although its clinical utility still requires further prospective validation.

## 1 Introduction

**Figure 1:**
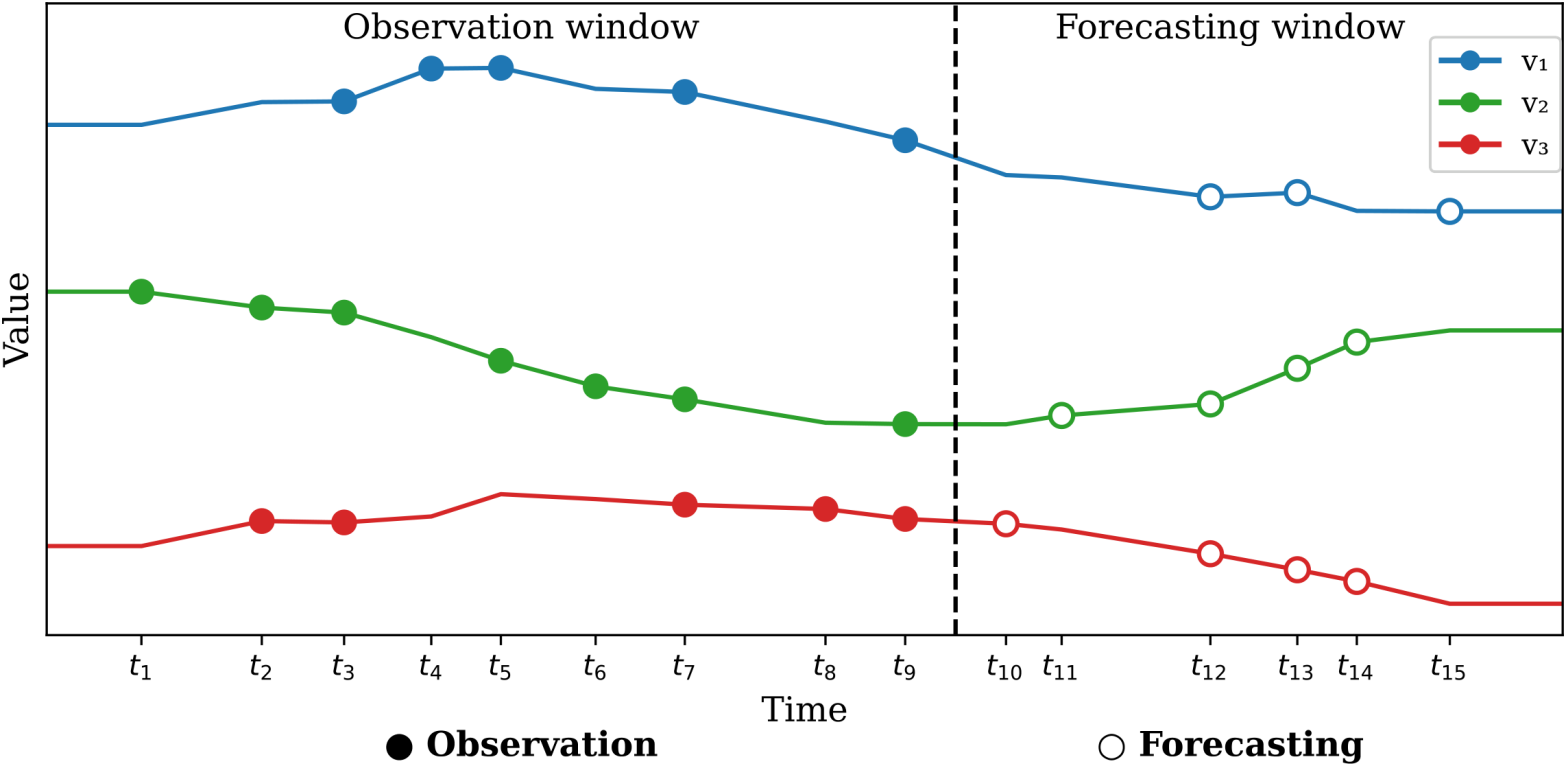
Irregular multivariate time series prediction problem, where v1, v2, and v3 represent three different variables.

As healthcare big data and artificial intelligence technologies continue to mature, in-depth analysis of time-series data captured in electronic health records (EHRs) has become a hot topic in current research. Medical time series data are ubiquitous in healthcare environments, ranging from electronic health records (EHRs) to continuous physiological monitoring in intensive care units (ICUs). These data streams contain critical information about patient status, disease progression, and treatment efficacy, making them invaluable for clinical decision-making. Unlike conventional time series, medical time series present unique challenges: they are often irregularly sampled, contain missing values, exhibit complex temporal dependencies, and demonstrate significant inter-variable correlations that evolve dynamically over time (Luo et al., 2024). Early prediction of physiological deterioration can enable timely interventions Kumaragurubaran et al., 2024). However, developing effective predictive models for irregular multivariate medical time series remains challenging due to their inherent complexity and the high stakes of medical applications. Traditional approaches to medical time series prediction have relied on statistical methods such as autoregressive integrated moving average (ARIMA) models (Singh et al., 2020). While these methods provide interpretable results, they often fail to capture the complex nonlinear relationships and long-term dependencies present in medical data. More recently, deep learning approaches have demonstrated promising results in handling the complexity of medical time series (Morid et al., 2023). Recurrent neural networks (RNNs) and their variants, particularly Long Short-Term Memory (LSTM) networks, have been widely applied to regular time series prediction tasks (Lindemann et al., 2021). However, these approaches typically require preprocessing steps like imputation or resampling to handle irregular sampling, potentially losing valuable information about the temporal dynamics of the original data. While Multivariate Time Series (MTS) forecasting is well studied, research predominantly focuses on regularly sampled and fully observed data (Lim and Zohren, 2021). Conversely, Irregular Multivariate Time Series (IMTS), characterized by non-uniform sampling intervals and missing observations, present unique predictive challenges that have received considerably less attention. In the field of medical informatics, one major focus remains continuous medical records, especially time series predictive modeling. With the successful application and rapid popularization of electronic health records (EHR), the amount of clinical medical time series data available for analysis has grown exponentially. As a result, medical health has gradually become one of the most promising frontiers in the field of data mining. Particularly within healthcare, accurate IMTS forecasting is fundamental for informed clinical decision-making and proactive planning. Modeling IMTS poses greater difficulties than regular MTS due to inherent intra-series irregularities and inter-series asynchrony (Horn et al., 2020). As illustrated in **Figure 1**, the IMTS prediction task involves forecasting values corresponding to specific query times based on historical observations. Although initial efforts have addressed IMTS prediction (Schirmer et al., 2022), many rely on Neural Ordinary Differential Equations (ODEs) (Chen et al., 2018). These approaches primarily tackle intra-series irregularities but often neglect crucial inter-series correlations and suffer from high computational costs associated with numerical integration, impacting both training and inference efficiency (Biloš et al., 2021).

Accurate IMTS forecasting faces three primary challenges: (1) Intra-series irregularity: Varying time intervals between consecutive observations disrupt data consistency, hindering the ability of conventional models (Lim and Zohren, 2021) to capture underlying temporal dynamics accurately (Rubanova et al., 2019). (2) Inter-series asynchrony: Observations across different variables in IMTS are often temporally misaligned due to irregular sampling or missing data. This misalignment complicates direct comparisons and correlation modeling, potentially obscuring true inter-series relationships. (3) Complex temporal patterns: Medical time series exhibit diverse periodic and non-periodic fluctuations that vary across patients and conditions. The clinical significance of these patterns is context-dependent and evolves, demanding models capable of adaptively assessing feature importance for accurate prediction. Most existing approaches still face limitations in simultaneously addressing the irregular sampling patterns, multi-scale temporal dependencies, and complex inter-variable relationships characteristic of medical time series data. Additionally, few methods effectively incorporate domain-specific knowledge about physiological systems and their periodicities, which could enhance prediction accuracy in medical contexts. To this end, we propose a new method for irregular multivariate medical time series prediction using Multi-scale Temporal-Frequency domain fusion Patching and Dynamic Graph modeling (MTFP-DG) for predicting medical IMTS. MTFP-DG firstly transforms each univariate irregular time series into a series of multi-scale patches, corresponding to each patch size. These patches differ in the number of observations but maintain a uniform time-scale resolution. This approach captures both fine short-term dynamics and macro long-term trends, enhances local semantic capture of irregular series, and naturally achieves patch-level temporal alignment, which lays the foundation for subsequent cross-series modeling and effectively circumvents the potential bias of forced pre-alignment. Along this line, dual-domain coding is introduced, combining a Transformable Time-aware Convolution Network (TTCN) with time-domain features and an Irregular Fourier Analysis Network (IFAN) with frequency-domain features, in order to encode each multi-scale patch as a latent embedding, which is subsequently used as an input token for a Temporal Transformer for intra-time-series dependency modeling. Based on this, a dynamical map combining Fourier coefficients with temporal embeddings was constructed for inter-time-series dependency modeling. In order to explicitly represent the dynamic correlations between IMTSs, we learn the corresponding time-varying adaptive graphs, which thus maintain the same temporal resolution as multi-scale patches. Then, graph neural networks are applied to these learnt graphs to model the patch-level dynamic correlations between IMTSs. Finally, final predictions are generated through the IFAN output layer. Accurately forecasting future values in irregular multivariate physiological time series may eventually support anticipatory monitoring and risk assessment in medical settings. However, the present study is designed as a methodological investigation of retrospective forecasting performance rather than a clinical effectiveness study. Accordingly, we frame MTFP-DG primarily as a modeling contribution for medical irregular multivariate time series, while considering possible clinical use as a downstream translational direction that requires additional prospective validation. Our main contributions are summarized as follows:

- To achieve both local semantic capture and cross-time-series correlation modeling of IMTS, a novel multi-scale patching method is proposed to convert each univariate irregular time series into a series of variable length but temporally aligned patches.
- We design a dual-domain fusion encoding mechanism that integrates temporal features with frequency features, enabling richer patch representations and providing more comprehensive semantic information for time series correlation modeling.
- We develop a dynamic graph learning module that constructs time-varying adaptive graphs and explicitly models cross-series dependencies for improved prediction accuracy.
- We introduce the interval-weighted loss specifically for medical prediction tasks, which enhances the clinical relevance of the predictions and improves the accuracy for long-term forecasting.

## 2 Related works

### 2.1 Irregular multivariate time series forecasting

Predicting irregular multivariate time series (IMTS), especially in healthcare, poses unique challenges due to measurement asynchrony, varying sampling frequencies of different variables, and inherent data sparsity. Such data are commonly found in electronic health records (EHRs) and intensive care units (ICUs), and are critical for timely clinical decisions, such as early warning of patient deterioration or prediction of disease progression. Current efforts regarding IMTS have mainly focused on classification tasks (Li et al., 2023; Liu et al., 2025a). Only a few forward-looking studies have made efforts in IMTS forecasting (Zhang et al., 2024a). Early attempts to apply machine learning involved feature engineering in combination with standard predictive models, but these methods are often ineffective in capturing complex temporal dynamics, especially in high-risk medical scenarios where subtle changes may signal significant clinical events. With the rise of deep learning, recurrent neural networks (RNNs), especially LSTMs and GRUs, have been applied to time series prediction. Like Phased LSTM (Neil et al., 2016) and GRU-D (Che et al., 2018) such improvements were proposed to explicitly deal with missing data and time gaps inherent in irregular medical sequences. For example, GRU-D, which utilizes a decay mechanism to interpolate missing values based on the last observation and elapsed time, has shown potential on clinical datasets. However, RNNs may face difficulties in capturing very long-term dependencies and may need to carefully handle the time gap between observations, which can be very variable in medical settings (e.g., lab tests vs. continuous vital signs monitoring). More recently, attention mechanisms and Transformer architectures have shown potential, but their direct application to raw irregular medical data often requires significant adaptation or preprocessing steps, which can sometimes introduce bias or obscure clinically relevant information. Although these studies have done a great deal of work in dealing with irregularities in irregular time series, how to effectively model correlations between time series in asynchronous medical IMTS (e.g., dynamic interactions between cardiovascular and respiratory signals) are still a problem to be explored. In addition, many existing models do not explicitly integrate mechanisms to deal with the multiscale nature of medical data, whereas both short-term fluctuations (e.g., heart rate variability) and long-term trends (e.g., gradual decline in renal function) are prognostically important.

### 2.2 Handling irregular sampling in time series

Resolving the irregular sampling characteristics of IMTS is a key prerequisite for effective modeling. A common approach is interpolation, where missing values are filled in to create a regular grid. Methods range from simple techniques (e.g., Last Observation Carry Forward (LOCF) or mean interpolation) to more complex statistical methods (e.g., Gaussian processes) (Schulz et al., 2018) and deep learning-based interpolation models (e.g., using auto-encoders or GANs) (Zhou et al., 2025). While interpolation makes the data compatible with the standard model, it may introduce artefacts or biases, especially if the irregularities themselves contain information, and may mask the true underlying dynamics (Lepot et al., 2017). Another transformation approach focuses on pre-aligned representations. This involves extending all univariate sequences in the IMTS to a uniform length corresponding to the set of all unique timestamps in all variables (Shukla and Marlin, 2021; Zhang et al., 2021; Schirmer et al., 2022). The goal is to achieve a temporal alignment suitable for subsequent modeling steps. However, this representation suffers from serious scalability issues, as the length of the sequence explodes as the number of variables or unique timestamps increases, leading to prohibitive computational and memory overheads. Horn et al (Horn et al., 2020). introduced a more scalable representation by treating IMTS observations as a set of tuples, including time, value, and variable indicators, and then aggregating these tuples are aggregated for IMTS classification. However, this representation may not be suitable for prediction tasks that require a more detailed and differentiated analysis of each variable. There are also methods designed to deal directly with irregular structures. Continuous-time models, such as Neural Ordinary Differential Equations (Neural ODEs) (Chen et al., 2018) and Latent ODEs (Rubanova et al., 2019), model the underlying dynamics as continuous processes inferred from sparse observations. These approaches are theoretically elegant, but can be computationally intensive and sensitive to hyperparameter selection. Other work focuses on time-aware adaptation of existing architectures. For example, temporal information (e.g., time elapsed since the last observation) can be used as an input feature or to regulate gating within an RNN (Al Olaimat et al., 2024) or attention mechanism (Xu et al., 2022). In addition, there are some approaches that divide time series into blocks at the sub-sequence level as input tokens for the Transformer (Nie et al., 2022). Liu et al (Liu et al., 2025b). designed a novel time-aware patch aggregation (TAPA) module to achieve adaptive patching. By learning dynamically adjustable patch boundaries and time-aware weighted averaging strategies, TAPA converts the original irregular sequence into a high-quality regularized representation in a channel-independent manner.

### 2.3 Modeling temporal and inter-variable dependencies

Capturing both the evolution of time series (temporal dependencies) and the complex interactions between different variables (inter-variable dependencies) is crucial for accurate IMTS prediction. For temporal dependencies, while RNNs were foundational, Transformer models (Han et al., 2021) have recently gained significant traction due to their effectiveness in capturing long-range dependencies via 164 self-attention mechanisms. Models like Informer (Zhou et al., 2021), Autoformer (Wu et al., 2021), and FEDformer (Zhou et al., 2022) introduced innovations like sparsity attention and frequency-domain processing to improve efficiency and performance for long sequence forecasting. Applying Transformers to time series often involves patching or tokenization, where the continuous series is segmented into patches that serve as input tokens (Wen et al., 2022), similar to image processing (e.g., ViT) or NLP. PatchTST (Nie et al., 2022) demonstrated the effectiveness of channel-independent patching for standard MTS. For inter-variable dependencies, multivariate extensions of RNNs or Transformers can implicitly learn cross-variable correlations. However, Graph Neural Networks (GNNs) (Wu et al., 2020) offer a more explicit and structured way to model these relationships, treating variables as nodes and their interactions as edges. GNNs have been successfully applied to MTS forecasting (Cui et al., 2021) by learning static or dynamic graph structures representing variable correlations. For instance, MTGNN (Gao et al., 2022) learns a static adjacency matrix, while models like Graph WaveNet (Wu et al., 2019) and StemGNN (Cao et al., 2020) capture dynamic spatial-temporal dependencies. DCRNN (Li et al., 2017) and STGCN (Yu et al., 2017), primarily relied on pre-defined graph structures to capture inter-variable relationships. However, obtaining such explicit structures is often infeasible in many domains. Consequently, subsequent research efforts focused on learning the graph structure directly from data, thereby automatically inferring the topological relationships between variables (Huang et al., 2023). Very recently, Deng et al (Deng et al., 2025). proposed a hypergraph network with multi-granularity temporal representation for traffic accident prediction, where hypergraph convolution is used to capture higher-order spatial relations and a multi-granularity temporal module is designed to jointly model short-term fluctuations and long-term trends. In parallel, Hou et al (Hou et al., 2025). developed a parallel multi-scale dynamic graph neural network for multivariate time series forecasting, which constructs dynamic graphs at multiple temporal scales and models them in a parallel manner to exploit scale-specific dependencies. These works highlight two emerging directions in graph-based temporal modeling: higher-order relational representations and multi-scale dynamic graph learning. Nevertheless, applying these GNN-based approaches to Irregular Multivariate Time Series (IMTS) introduces significant challenges. The inherent asynchrony and temporal misalignment of observations across different variables complicate the modeling of inter-series correlations. One attempt to address this is Raindrop (Zhang et al., 2021), which propagates asynchronous observations across all variables at the timestamp of any observation’s occurrence. This method, however, necessitates a form of pre-alignment for the IMTS data and potentially suffers from the issue of sequence length explosion. Another related line of research involves applying GNNs to model regular Multivariate Time Series (MTS) with missing data (Chen et al., 2023). These approaches typically require aligning the MTS with missing values at specific moments, akin to the aforementioned pre-alignment strategies, and their primary focus is on handling data imputation or loss rather than the fundamental irregularity and asynchrony. In contrast, our work diverges from these paradigms by emphasizing the circumvention of canonical pre-alignment representations, aiming to directly address the challenges posed by irregularity and asynchrony in modeling IMTS.

Another research line for modeling the relational dependencies among variables in medical multivariate time series lies in investigating physiological interactions from the perspective of network physiology. Network Physiology emphasizes that physiological states emerge from dynamic coordination and network interactions among coupled organ systems and subsystems rather than from isolated variables alone (Ivanov, 2021). In this broader context, interactions among physiological variables have been investigated using multiple methodological perspectives, including statistical and information-theoretic approaches. For example (Antonacci et al., 2020), quantified information transfer and interaction topology in multivariate physiological processes using penalized regression within a VAR/state-space framework. More recently, interpretable machine-learning approaches have also been used to characterize higher-order interaction patterns in physiological regulation; for instance (Ontivero-Ortega et al., 2025), analyzed sex-related differences in cardiovascular control through high-order feature importance derived from heart-rate and blood-pressure variability signals. These studies are closely related in scientific motivation, as they all aim to better understand cross-variable physiological coupling. Our research aims to construct a task-oriented latent relational structure that support prediction and summarize time-varying associative dependencies.

## 3 Preliminaries and problem formulation

### 3.1 Definition of Irregular Multivariate Time Series

An Irregular Multivariate Time Series (IMTS) dataset represents observations from multiple ( N>1) time-dependent variables or channels. Let $X=\{X_{1},X_{2},\ldots ,X_{N}\}$ denote such a dataset, where each $X_{i}$ ( 1≤i≤N) corresponds to a single variable. Each $X_{i}$ is represented as a time-ordered sequence of observation tuples (Equation 1):

(1) $$X_{i}=\{(t_{i,1},v_{i,1}),(t_{i,2},v_{i,2}),\ldots ,(t_{i,M_{i}},v_{i,M_{i}})\}$$

Here, $\left(t_{i,j},v_{i,j}\right)$ is the *j*-th observation for variable *i*, consisting of a timestamp $t_{i,j}\in {\mathbb{R}}^{+}$ (where $t_{i,j}<t_{i,j+1}$) and a corresponding value $v_{i,j}\in {\mathbb{R}}^{D}$ (often D=1). $M_{i}$ denotes the total number of observations for variable *i*. IMTS are characterized by:

- Irregular Timestamps: The time intervals $\text{Δ}t_{i,j}=t_{i,j+1}-t_{i,j}$ within a single variable series $X_{i}$ are generally not constant.
- Asynchronous Observations: The sets of timestamps $\left\{t_{i,1},\ldots ,t_{i,M_{i}}\right\}$ usually differ across distinct variables i≠k.

This structure is common in healthcare settings, like Electronic Health Records (EHR), where different clinical measurements are taken at varying and often unsynchronized times.

### 3.2 IMTS forecasting task

The objective of this work is to forecast future values of an IMTS, specifically for medical diagnostic prediction. Given the history of all observations up to a time *T*, denoted as $X_{\left[0,T\right]}=\{(t_{i,j},v_{i,j})❘1\leq i\leq N,0\leq t_{i,j}\leq T\}$, we aim to predict the values of all *N* variables over a future prediction horizon of length *H*. Specifically, we focus on predicting values at $H^{\prime }$ discrete future time points $\tau =\{\tau_{1},\tau_{2},\ldots ,\tau_{H^{\prime }}\}$, where $T<\tau_{1}<\tau_{2}<\ldots <\tau_{H^{\prime }}\leq T+H$. Formally, we seek to learn a predictive model *f* that maps the historical observations to the future predictions (Equation 2):

(2) $$f:X_{\left[0,T\right]}\to \hat{Y}$$

The output $\hat{Y}=\{{\hat{y}}_{i,k}\in {\mathbb{R}}^{D}❘i=1,\ldots ,N;k=1,\ldots ,H^{\prime }\}$ is the set of predicted values, where ${\hat{y}}_{i,k}$ is the prediction for variable *i* at future time $\tau_{k}$. The model *f* is optimized by minimizing a loss function $L(Y,\hat{Y})$ that measures the difference between the predictions $\hat{Y}$ and the corresponding ground truth future values $Y=\{y_{i,k}❘i=1,\ldots ,N;k=1,\ldots ,H^{\prime }\}$. This paper proposes the MTFP-DG model as the function *f*, utilizing specialized loss functions designed for medical IMTS.

## 4 Materials and methods

**Figure 2:**
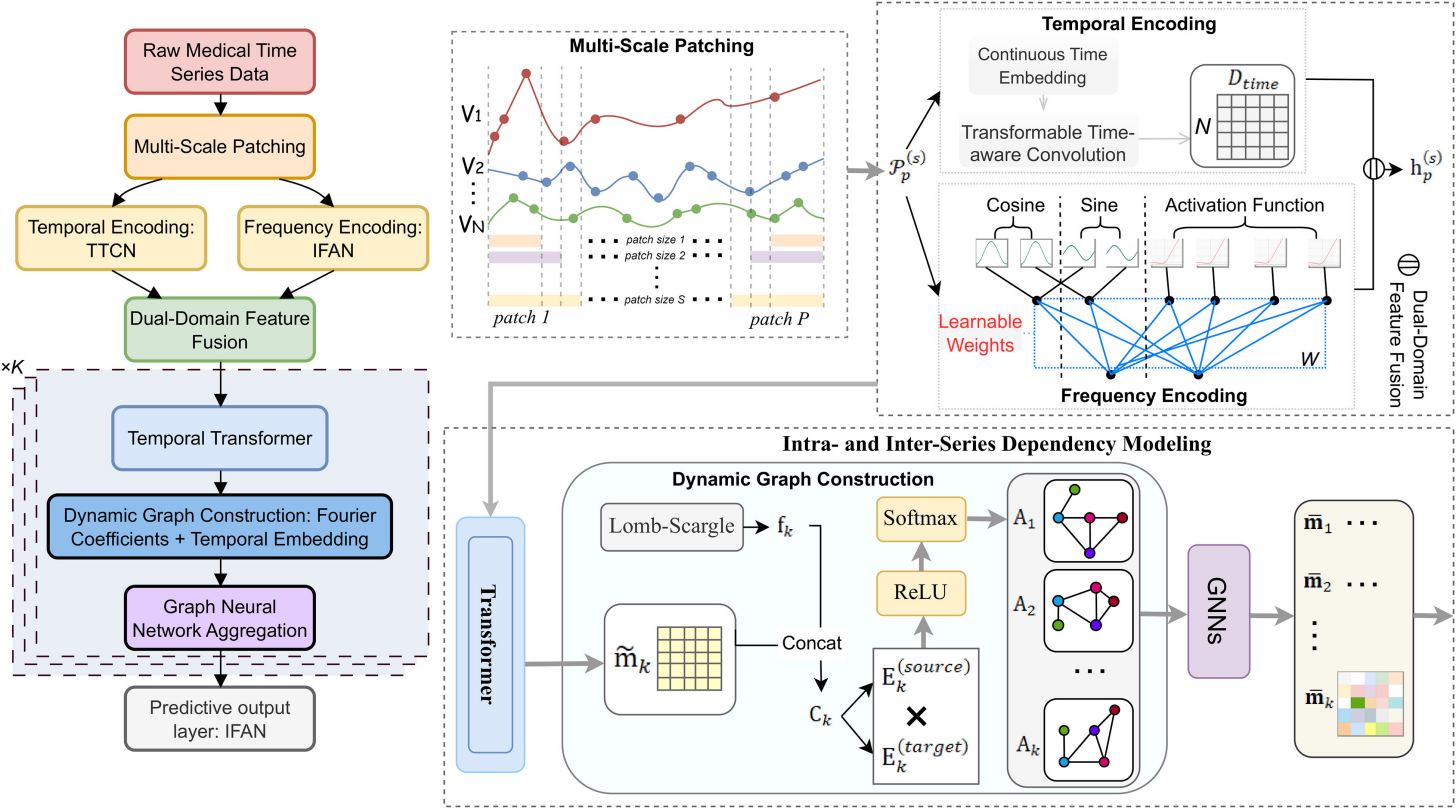
Overview of the proposed MTFP-DG framework for irregular multivariate time series (IMTS) forecasting. Left: the pipeline (multi-scale patching → dual-domain encoding via Transformable Time-aware Convolution Network (TTCN) and Irregular Fourier Analysis Networks (IFAN) → Temporal Transformer → interval-wise dynamic graphaggregation → IFAN prediction head). Right: expanded views of the main modules.

An overview of MTFP-DG is shown in **Figure 2**. The method incorporates Multi-scale Patching techniques to deal with irregularities and capture diverse temporal patterns, uses temporal-frequency dual-domain feature coding to combine time and frequency information, employs the Transformer module to deal with intra-sequence dependencies, and models inter-sequence correlations via a dynamic graph neural network. In the subsequent sections, we present the technical details of the irregular time series multiscale patching, dual-domain patch embedding, intra-sequence dependency modeling, inter-sequence dependency modeling, and IMTS prediction process in turn.

### 4.1 Multi-scale patching for IMTS

Conventional time series imputation methodologies typically segment regularly-sampled temporal sequences into a series of patch-level imputations, each comprising a predefined number of consecutive observations. However, within the context of Irregular Multivariate Time Series (IMTS), this approach engenders significant methodological limitations due to heterogeneous temporal intervals between observations. The application of fixed-cardinality patching to IMTS data inherently produces patches that span highly variable temporal domains. For instance, a patch constructed from five sequential observations might encompass merely minutes in densely-sampled regions but extend across days in sparsely-sampled regions. This substantial variability in patch temporal resolution further exacerbates the intrinsic irregularity and asynchrony challenges already prevalent in IMTS modeling. Such temporal inconsistency fundamentally undermines the ability to establish meaningful cross-patch comparisons and significantly hinders the model’s capacity to capture consistent temporal dynamics across varying sampling densities. We propose Multi-Scale Patching for IMTS. Given an input IMTS $X_{\left[0,T\right]}$, we first process each univariate series $X_{i}=\{(t_{i,j},v_{i,j})❘0\leq t_{i,j}\leq T\}$ independently. We define *S* different patch time duration scales, $L_{1},L_{2},\ldots ,L_{S}$. For each scale s∈{1,…,S}, we partition each time axis [0,T] into $P_{s}=\lfloor T/L_{s}\rfloor$ non-overlapping intervals (patches), where the *p*-th interval is $\left[(p-1)L_{s},pL_{s}\right]$. A patch $P_{i,p}^{\left(s\right)}$ for variable *i* at scale *s* and time interval *p* comprises all observations falling within this time window (Equation 3):

(3) $$P_{i,p}^{\left(s\right)}=\{(t_{i,j},v_{i,j})❘(p-1)L_{s}\leq t_{i,j}<pL_{s}\}$$

**Figure 3:**
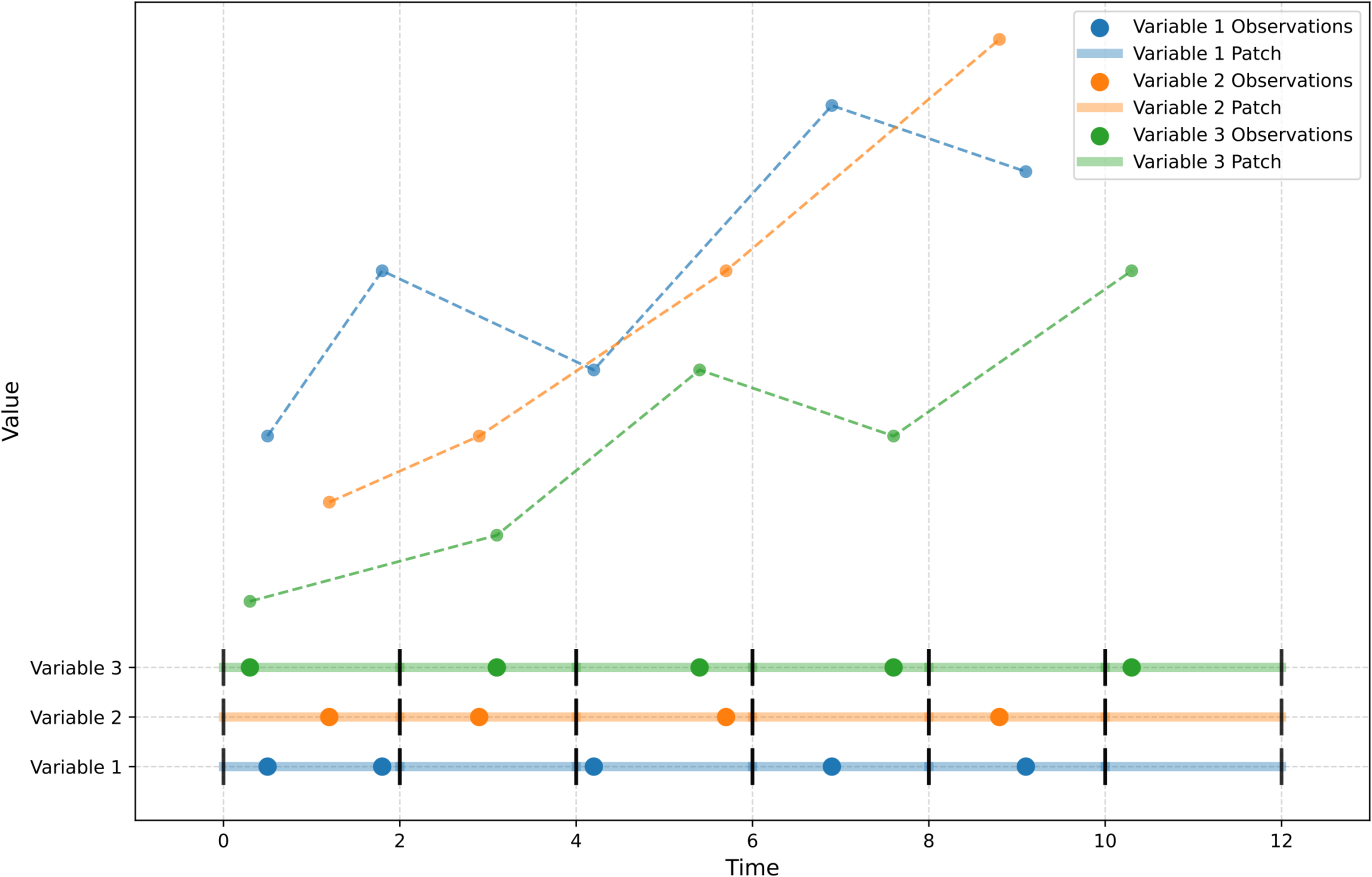
Conceptual diagram of patches for a given patch size (e.g., 2 hours) over a given period of time. (Each patch is separated by a short vertical line.).

Note that the number of observations $❘P_{i,p}^{\left(s\right)}❘$ varies across different variables *i* and intervals *p* due to the irregular sampling. All patches $\left\{P_{1,p}^{\left(s\right)},\ldots ,P_{N,p}^{\left(s\right)}\right\}$ corresponding to the same interval index *p* and scale *s* share the exact same time duration $L_{s}$ and cover the same time window $\left[(p-1)L_{s},pL_{s}\right]$. **Figure 3** is a concept diagram showing patches of a patch size (e.g., 2 hours) over a certain period of time, with each patch spanning a patch window size with a uniform time range to ensure consistent temporal resolution across time and variables. This inherent temporal alignment at the patch level avoids the need for potentially biased pre-alignment or interpolation of the raw data.

The motivation for using multiple patch durations is to explicitly balance a key trade-off in medical irregular time series. Very small patches preserve transient local changes, but under sparse and asynchronous sampling they may contain too few observations to form a stable local representation. In contrast, larger patches are more likely to contain sufficient observations and are better suited to summarizing coarse trends, but they may smooth out short-lived physiological fluctuations. The multi-scale design is therefore intended to avoid committing the model to a single temporal resolution and to allow the model to simultaneously capture fine-grained local dynamics (using small $L_{s}$) and coarse-grained long-term trends (using large $L_{s}$).

### 4.2 Dual-domain patch embedding

Following the transformation of each univariate irregular time series into a series of multi-scale patches, each patch is encoded as a latent embedding in order to capture the local semantics in the time series. For each non-empty patch $P_{i,p}^{\left(s\right)}$, we generate a latent embedding $h_{i,p}^{\left(s\right)}$ by fusing features from both the time and frequency domains.

#### 4.2.1 Temporal encoding

To effectively extract dynamic temporal features and local semantic information from each multi-scale patch $P_{i,p}^{\left(s\right)}$, we adopt a Transformable Time-aware Convolution Network (TTCN) (Zhang et al., 2024b). This network is specifically designed to process sequences of observations characterized by variable lengths and irregular internal sampling, as found within our patches. For each patch $P_{i,p}^{\left(s\right)}=\{(t_{i,j,p}^{\left(s\right)},v_{i,j,p}^{\left(s\right)})❘j=1,\ldots ,L_{i,p}^{\left(s\right)}\}$, where $L_{i,p}^{\left(s\right)}$ denotes the number of observations within that patch for variable *i* at scale *s* and interval *p*, we first augment the raw observations. The timestamps $t_{i,j,p}^{\left(s\right)}$ are encoded using a continuous time embedding function ϕ(t), which typically combines linear and periodic components to capture both non-periodic trends and cyclical patterns (Equation 4):

(4) $$\phi (t)[d]=\{\begin{array}{ll} \omega_{0}\cdot t+\alpha_{0}, & \mathrm{if}\;d=0\\ \text{sin}(\omega_{d}\cdot t+\alpha_{d}), & \text{if}\;0<d<D_{t} \end{array}$$

where $\omega_{d}$ and $\alpha_{d}$ are learnable frequency and phase parameters, respectively, and $D_{t}$ is the dimensionality of the time embedding. This time embedding $\phi (t_{i,j,p}^{\left(s\right)})$ is then concatenated with the original observation value $v_{i,j,p}^{\left(s\right)}\in {\mathbb{R}}^{D_{v}}$ (where $D_{v}$ is the dimensionality of the observation value) to form an augmented feature representation $z_{j,i,p}^{\left(s\right)}\in {\mathbb{R}}^{D_{in}}$ for each point within the patch, where $D_{in}=D_{t}+D_{v}$. For reproducibility, we explicitly specify the timestamp rescaling and the concatenation order used to form the augmented input $z_{j,i,p}^{\left(s\right)}$. Given a patch interval $\left[(p-1)L_{s},pL_{s}\right)$ at scale *s*, we linearly rescale each timestamp within the patch by (Equation 5)

(5) $${\tilde{t}}_{i,j,p}^{\left(s\right)}=\frac{t_{i,j,p}^{\left(s\right)}-(p-1)L_{s}}{L_{s}}\in [0,1),$$

and the continuous time embedding is computed as $\phi ({\tilde{t}}_{i,j,p}^{\left(s\right)})\in {\mathbb{R}}^{D_{t}}$. We then form the augmented feature by concatenating (Equation 6):

(6) $$z_{j,i,p}^{\left(s\right)}=[\phi ({\tilde{t}}_{i,j,p}^{\left(s\right)})\;∥\;v_{i,j,p}^{\left(s\right)}]\in {\mathbb{R}}^{D_{in}},\;D_{in}=D_{t}+D_{v},$$

where ∥ denotes concatenation along the feature dimension. The core mechanism of the TTCN involves a meta-filter $F_{k}$, which adaptively generates a time-aware convolutional filter, $f_{k,i,p}^{\left(s\right)}$ for each desired output feature map *k*. A key characteristic is that the size of this generated filter dynamically transforms to match the actual number of observations, $L_{i,p}^{\left(s\right)}$ within the current patch $P_{i,p}^{\left(s\right)}$. For the *k*-th output feature map, the corresponding derived convolutional filter $f_{k,i,p}^{\left(s\right)}\in {\mathbb{R}}^{L_{i,p}^{\left(s\right)}\times D_{in}}$ is formulated as (Equation 7):

(7) $$f_{k,i,p}^{\left(s\right)}[j]=\frac{\text{exp}(F_{k}(z_{j,i,p}^{\left(s\right)}))}{\sum_{m=1}^{L_{i,p}^{\left(s\right)}}\text{exp}(F_{k}(z_{m,i,p}^{\left(s\right)}))}$$

where $f_{k,i,p}^{\left(s\right)}[j]\in {\mathbb{R}}^{D_{in}}$ represents the filter weights corresponding to the *j*-th observation in the patch for the *k*-th meta-filter. In our implementation, each meta-filter $F_{k}(\cdot )$ is instantiated as a shared two-layer MLP (Equation 8):

(8) $$s_{k,i,p}^{\left(s\right)}[j]=F_{k}(z_{j,i,p}^{\left(s\right)})=W_{k,2}\mathrm{GELU}(W_{k,1}z_{j,i,p}^{\left(s\right)}+b_{k,1})+b_{k,2},$$

where $W_{k,1}\in {\mathbb{R}}^{d_{h}\times D_{in}}$, $W_{k,2}\in {\mathbb{R}}^{D_{in}\times d_{h}}$, and $d_{h}$ is the hidden size. The exponential and division in (Equation 7) are applied element-wise, and the normalization is a *channel-wise softmax over the observation index* within the patch. This makes the normalization axis explicit (over *j* for each feature channel *d*) and ensures that $\sum_{j=1}^{L_{i,p}^{\left(s\right)}}f_{k,i,p}^{\left(s\right)}[j,d]=1$ for every *d*. This normalization is applied across the temporal dimension of the patch, ensuring consistent scaling of convolution results irrespective of sequence length. With $D_{\text{time}}$ such distinct derived filters (corresponding to the desired dimensionality of the temporal embedding), the TTCN performs a temporal convolution over the sequence of augmented features ${\left\{z_{j,i,p}^{\left(s\right)}\right\}}_{j=1}^{L_{i,p}^{\left(s\right)}}$ within the patch. The resulting temporal embedding $h_{i,p}^{\left(s,\text{time}\right)}\in {\mathbb{R}}^{D_{\text{time}}}$ is computed as (Equation 9):

(9) $$h_{i,p}^{\left(s,\text{time}\right)}[k]=\sum_{j=1}^{L_{i,p}^{\left(s\right)}}{\left(f_{k,i,p}^{\left(s\right)}[j]\right)}^{⊤}z_{j,i,p}^{\left(s\right)}$$

where $h_{i,p}^{\left(s,\text{time}\right)}[k]$ is the *k*-th element of the output temporal embedding. Handle irregularly spaced time intervals within patches, as the meta-filter learns from time-augmented input features. Efficiently model sequences of arbitrary length without requiring an increased number of learnable parameters for the filters themselves, as they are dynamically generated. In practice, TTCN’s transformable, timestamp-conditioned filters make patch embeddings explicitly gap-aware without increasing parameters with length. This yields stable representations under uneven sampling and high missingness. The output $h_{i,p}^{\left(s,\text{time}\right)}$ serves as the comprehensive temporal representation of the patch $P_{i,p}^{\left(s\right)}$, which is subsequently fused with its frequency-domain counterpart.

For a non-empty patch $P_{i,p}^{\left(s\right)}={\left\{(t_{i,j,p}^{\left(s\right)},v_{i,j,p}^{\left(s\right)})\right\}}_{j=1}^{L_{i,p}^{\left(s\right)}}$, TTCN is computed as:

1. Timestamp rescaling: compute ${\tilde{t}}_{i,j,p}^{\left(s\right)}=\frac{t_{i,j,p}^{\left(s\right)}-(p-1)L_{s}}{L_{s}}$ for all *j*.
2. Time embedding: compute $\phi ({\tilde{t}}_{i,j,p}^{\left(s\right)})\in {\mathbb{R}}^{D_{t}}$.
3. Feature concatenation: form $z_{j,i,p}^{\left(s\right)}=[\phi ({\tilde{t}}_{i,j,p}^{\left(s\right)})∥v_{i,j,p}^{\left(s\right)}]\in {\mathbb{R}}^{D_{in}}$.
4. Meta-filter scoring: for each output channel $k\in \{1,\ldots ,D_{\text{time}}\}$, compute $s_{k,i,p}^{\left(s\right)}[j]=F_{k}(z_{j,i,p}^{\left(s\right)})\in {\mathbb{R}}^{D_{in}}$.
5. Softmax normalization over observations: compute $f_{k,i,p}^{\left(s\right)}[j,d]$ by channel-wise softmax over *j*.
6. Weighted aggregation (transformable convolution): compute $h_{i,p}^{\left(s,\text{time}\right)}[k]=\sum_{j=1}^{L_{i,p}^{\left(s\right)}}{\left(f_{k,i,p}^{\left(s\right)}[j]\right)}^{⊤}z_{j,i,p}^{\left(s\right)}$.

#### 4.2.2 Frequency encoding

To extract frequency-domain features from time series patches $P_{i,p}^{\left(s\right)}$ characterized by irregular sampling, we adapt Fourier Analysis Networks (FAN) (Dong et al., 2024), which was originally developed for regularly sampled sequences, and refer to the resulting irregular-time variant as an Irregular Fourier Analysis Network (IFAN). The IFAN module processes each observation $\left(t_{j},v_{j}\right)$ within a patch $P_{i,p}^{\left(s\right)}=\{(t_{i,j,p}^{\left(s\right)},v_{i,j,p}^{\left(s\right)})❘j=1,\ldots ,L_{i,p}^{\left(s\right)}\}$ (indices i,p,s are omitted temporarily for conciseness in defining the core operation). Here, $v_{j}\in {\mathbb{R}}^{D_{v}}$ represents the observation value at the current time point, serving as input x, and $t_{j}\in \mathbb{R}$ is its corresponding normalized timestamp. The fundamental computational unit within the IFAN module, the time-aware Fourier analysis layer ϕ(x,t), is defined as (Equation 10):

(10) $$\phi (x,t)=[\text{cos}(W_{p}x+U_{p}t)\;∥\;\text{sin}(W_{p}x+U_{p}t)\;∥\;\sigma (B_{\bar{p}}+W_{\bar{p}}x+V_{\bar{p}}t)]$$

In this formulation, the parameters are defined as: $W_{p}\in {\mathbb{R}}^{d_{p}\times D_{v}}$ is a learnable weight matrix that linearly transforms the observation value *x* for the periodic components of the Fourier series. $U_{p}\in {\mathbb{R}}^{d_{p}\times 1}$ is a learnable weight vector for the linear influence of the timestamp *t* within these periodic components, serving to adjust their phase and frequency response. Correspondingly, $W_{\bar{p}}\in {\mathbb{R}}^{d_{\bar{p}}\times D_{v}}$ is a learnable weight matrix that linearly transforms *z* for the non-linear feature extraction component. $V_{\bar{p}}\in {\mathbb{R}}^{d_{\bar{p}}\times 1}$ is a learnable weight vector for the influence of *t* in this non-linear part, designed to enhance the model’s adaptability to temporal irregularities. $B_{\bar{p}}\in {\mathbb{R}}^{d_{\bar{p}}}$ represents the bias vector for the non-linear component. The symbols $d_{p}$ and $d_{\bar{p}}$ denote the output feature dimensions of the Fourier periodic components and the non-linear component, respectively. The ∥ operator signifies vector concatenation, and σ is a non-linear activation function, specified as GELU. By explicitly incorporating the time *t* into the arguments of the cos(·) and sin(·) functions ( $W_{p}x+U_{p}t$) and into the input of the non-linear activation function ( $W_{\bar{p}}x+V_{\bar{p}}t$), the IFAN layer enables its frequency sensitivity and non-linear response to dynamically adjust based on the specific timing of each observation. Because the harmonics are directly modulated by timestamps, IFAN retains sensitivity to oscillatory signatures without committing to a surrogate regular grid, complementing TTCN’s gap-aware local dynamics.

A complete IFAN module is constructed by stacking $L_{\text{IFAN}}$ such time-aware Fourier analysis layers. Each observation-timestamp pair $\left(t_{j},v_{j}\right)$ within the input patch $P_{i,p}^{\left(s\right)}$ is processed independently by these $L_{\text{IFAN}}$ layers, which share parameters across all observations in the patch. This process yields a sequence of feature vectors, ${\left\{\phi_{L_{\text{IFAN}}}(t_{i,j,p}^{\left(s\right)},v_{i,j,p}^{\left(s\right)})\right\}}_{j=1}^{L_{i,p}^{\left(s\right)}}$, one for each observation within the patch. To obtain a single frequency embedding $h_{i,p}^{\left(s,\text{freq}\right)}$ representative of the entire patch $P_{i,p}^{\left(s\right)}$, an aggregation operation is performed on this sequence of feature vectors. Specifically, average pooling is employed (Equation 11):

(11) $$h_{i,p}^{\left(s,\text{freq}\right)}=\frac{1}{L_{i,p}^{\left(s\right)}}\sum_{j=1}^{L_{i,p}^{\left(s\right)}}\phi_{L_{\text{IFAN}}}(t_{i,j,p}^{\left(s\right)},v_{i,j,p}^{\left(s\right)})$$

This aggregation step ensures that the IFAN module outputs a fixed-dimension representation rich in the patch’s intrinsic frequency information, regardless of the number of observations within the patch. The design of IFAN ingeniously combines the periodic modeling capabilities of Fourier analysis with a direct adaptation to irregular timestamps by integrating observation values and their precise timings within its core layers. This point-wise processing followed by aggregation allows for the effective capture of frequency characteristics from irregularly sampled time series segments.

For each observation-timestamp pair $\left(t_{j},v_{j}\right)$ in a patch, IFAN applies $L_{\text{IFAN}}$ shared time-aware Fourier layers point-wise and then aggregates:

1. Timestamp rescaling: use the same rescaled timestamp ${\tilde{t}}_{j}\in [0,1)$ as defined in the TTCN subsection.

2. Point-wise transformation (layer l): let $x_{j}^{\left(0\right)}=v_{j}$. For $l=1,\ldots ,L_{\text{IFAN}}$, compute


$$u_{p,j}^{\left(l\right)}=W_{p}^{\left(l\right)}x_{j}^{\left(l-1\right)}+U_{p}^{\left(l\right)}{\tilde{t}}_{j},\;g_{j}^{\left(l\right)}=\text{GELU}(B_{\bar{p}}^{\left(l\right)}+W_{\bar{p}}^{\left(l\right)}x_{j}^{\left(l-1\right)}+V_{\bar{p}}^{\left(l\right)}{\tilde{t}}_{j}),$$

3. and update

4. $x_{j}^{\left(l\right)}=[\text{cos}(u_{p,j}^{\left(l\right)})\;∥\;\text{sin}(u_{p,j}^{\left(l\right)})\;∥\;g_{j}^{\left(l\right)}].$5. Patch-level aggregation: after $L_{\text{IFAN}}$ layers, denote $\phi_{L_{\text{IFAN}}}(t_{j},v_{j})=x_{j}^{\left(L_{\text{IFAN}}\right)}$ and compute the patch frequency embedding by mean pooling: $h_{i,p}^{\left(s,\text{freq}\right)}=\frac{1}{L_{i,p}^{\left(s\right)}}\sum_{j=1}^{L_{i,p}^{\left(s\right)}}\phi_{L_{\text{IFAN}}}(t_{i,j,p}^{\left(s\right)},v_{i,j,p}^{\left(s\right)})$.

This makes explicit that the timestamp enters both the periodic branch via $U_{p}^{\left(l\right)}{\tilde{t}}_{j}$ and the non-linear branch via $V_{\bar{p}}^{\left(l\right)}{\tilde{t}}_{j}$, while parameters are shared across all observations within the patch.

#### 4.2.3 Dual-domain feature fusion

Subsequent to the parallel extraction of temporal and frequency domain characteristics from each patch $P_{i,p}^{\left(s\right)}$, a fusion mechanism is employed to integrate these distinct representations into a unified and more comprehensive embedding. This dual-domain fusion aims to harness the complementary strengths of both temporal dynamics captured by the Time-varying Convolutional Network (TTCN) and frequency characteristics identified by the Irregular Fourier Analysis Networks (IFAN). Although the period term in the continuous time embedding ϕ(t) provides some time-periodicity sensing capability, it mainly encodes the ‘time axis’. The dedicated frequency domain feature extraction module analyses and encodes the intrinsic frequency structure of the ‘signal value’ over the time period. Temporal ordering information alone is not always sufficient to characterize the structure of physiological signals. The TTCN branch mainly emphasizes local temporal evolution, irregular gaps, and short-range semantic patterns within each patch, whereas many medical variables may also exhibit oscillatory or quasi-periodic behavior whose signatures are more naturally summarized in the spectral domain. Moreover, because the observations are irregularly sampled, directly applying regular-grid frequency transforms would require interpolation or resampling. In this sense, TTCN and IFAN are designed to be complementary: the former focuses on gap-aware temporal dynamics, while the latter emphasizes patch-level periodicity and frequency-selective response.

The inputs to this fusion stage are the temporal embedding $h_{i,p}^{\left(s,\text{time}\right)}\in {\mathbb{R}}^{D_{\text{time}}}$ derived from TTCN, and the frequency embedding $h_{i,p}^{\left(s,\text{freq}\right)}\in {\mathbb{R}}^{D_{\text{freq}}}$ produced by IFAN. To combine these features, we first perform a concatenation operation (Equation 12):

(12) $$h_{i,p}^{\left(s,\text{concat}\right)}=\text{Concat}(h_{i,p}^{\left(s,\text{time}\right)},h_{i,p}^{\left(s,\text{freq}\right)})$$

This results in an intermediate combined vector $h_{i,p}^{\left(s,\text{concat}\right)}\in {\mathbb{R}}^{D_{\text{time}}+D_{\text{freq}}}$, which amalgamates information from both domains. To allow for learned interactions between the concatenated features and to project the combined representation into a potentially different embedding space of dimension $D_{\text{fused}}$, this concatenated vector is then passed through a linear projection layer. This transformation is defined by (Equation 13):

(13) $$h_{i,p}^{\left(s\right)}=W_{\text{fusion}}h_{i,p}^{\left(s,\text{concat}\right)}+b_{\text{fusion}}$$

where $W_{\text{fusion}}\in {\mathbb{R}}^{D_{\text{fused}}\times (D_{\text{time}}+D_{\text{freq}})}$ is a learnable weight matrix, and $b_{\text{fusion}}\in {\mathbb{R}}^{D_{\text{fused}}}$ is a learnable bias vector. The resulting vector $h_{i,p}^{\left(s\right)}\in {\mathbb{R}}^{D_{\text{fused}}}$ constitutes the final fused dual-domain patch embedding. This fused embedding $h_{i,p}^{\left(s\right)}$ encapsulates a richer set of information by leveraging both the temporal evolution and the underlying periodic characteristics of the observations within the patch. It subsequently serves as the primary input token for the downstream intra-series dependency modeling components, such as the Transformer architecture.

### 4.3 Intra-series dependency modeling via transformer

Following the generation of dual-domain patch embeddings $h_{i,p}^{\left(s\right)}\in {\mathbb{R}}^{D_{\text{fused}}}$ for each patch *p* of variable *i* at scale *s*, the MTFP-DG model proceeds to capture temporal dependencies across these patch representations while concurrently integrating information derived from the *S* different scales. This crucial step ensures that both fine-grained, short-term dynamics and broader, long-term trends are synergistically utilized. The process unfolds by first explicitly integrating multi-scale features into a unified sequence for each variable, followed by temporal contextualization and deep multi-scale fusion using a Transformer architecture. Multi-Scale Feature Integration: The initial multi-scale patching and dual-domain encoding yield scale-specific patch embeddings $h_{i,p}^{\left(s\right)}.$ To create a unified representation that incorporates insights from all *S* scales for subsequent temporal modeling, we align and fuse these embeddings onto a canonical set of $P_{1}$ time intervals, defined by the finest patch scale (i.e., s=1, with patchduration $L_{1}$). For each variable *i* and each canonical time interval index $k\in \{1,\ldots ,P_{1}\}$, an integrated multi-scale feature vector is constructed. This involves aligning the patch embeddings from all *S* scales to this canonical timeline. Specifically, for each scale $s^{\prime }$, the patch embedding $h_{i,p}^{\left(s^{\prime }\right)}$ whose temporal span corresponds to or covers the *k*-th canonical interval is selected. This results in a set of *S* feature vectors $\left\{h_{i,k}^{*(1)},h_{i,k}^{*(2)},\ldots ,h_{i,k}^{*(S)}\right\}$ for variable *i* at canonical interval *k*, where each $h_{i,k}^{*(s^{\prime })}\in {\mathbb{R}}^{D_{\text{fused}}}$ is the dual-domain embedding from scale $s^{\prime }$ pertinent to interval *k*. These *S* scale-specific embeddings are then concatenated to form $h_{i,k}^{\left(\text{ms}-\text{concat}\right)}=\text{Concat}(h_{i,k}^{*(1)},\ldots ,h_{i,k}^{*(S)})$. This vector is subsequently projected through a linear layer with a GELU activation function (Equation 14):

(14) $$m_{i,k}=\text{GELU}(W_{\text{ms}-\text{fusion}}h_{i,k}^{\left(\text{ms}-\text{concat}\right)}+b_{\text{ms}-\text{fusion}})$$

where $W_{\text{ms}-\text{fusion}}\in {\mathbb{R}}^{D_{\text{input}\_\text{tf}}\times (S\cdot D_{\text{fused}})}$ and $b_{\text{ms}-\text{fusion}}\in {\mathbb{R}}^{D_{\text{input}\_\text{tf}}}$ are learnable parameters, yielding the integrated multi-scale representation $m_{i,k}\in {\mathbb{R}}^{D_{\text{input}\_\text{tf}}}$. Each $m_{i,k}$ now serves as a rich token for variable *i* at canonical time interval *k*, inherently carrying explicitly aggregated multi-scale information. Transformer-based Temporal Contextualization and Deep Multi-Scale Fusion: The sequence of these integrated multi-scale representations $\left(m_{i,1},m_{i,2},\ldots ,m_{i,P_{1}}\right)$ for each variable *i* (denoted as $M_{i,1:P_{1}}$) serves as input tokens tothe Temporal Transformer (i.e., a standard Transformer encoder applied to canonical-interval tokens to model dependencies across successive intervals.). This module operates independently on each variable’s sequence, adhering to the standard encoder architecture. Positional encodings $PE_{1:P_{1}}\in {\mathbb{R}}^{P_{1}\times D_{\text{input}\_\text{tf}}}$ are added to the input embeddings $M_{i,1:P_{1}}$ to provide the model with information about the sequential order of these canonical time intervals. The Transformer encoder, composed of stacked layers of multi-head self-attention (MHSA) and position-wise feed-forward networks (FFN), facilitated by residual connections and layer normalization, then processes this sequence. The MHSA mechanism is pivotal not only for capturing temporal dependencies between different time intervals *k* but also for performing a deeper, context-aware fusion of the multi-scale features already embedded within each $m_{i,k}$. As each $m_{i,k}$ contains an explicit aggregation of features from different scales (representing both short-term and long-term characteristics pertinent to that interval), the self-attention mechanism computes attention scores across the entire sequence of these multi-scale tokens. In doing so, it learns to dynamically weigh the importance of different time steps. Crucially, because each token is already multi-scale, the attention mechanism implicitly learns how different scales of information at one time step relate to or influence other (potentially multi-scale) information at other time steps. The model learns, in different temporal contexts, which aspects of the pre-fused multi-scale information (i.e., which embedded scales within $m_{i,k}$) are most salient for predicting future states, thereby achieving a sophisticated and adaptive integration of short-term dynamics and long-term trends. For each attention head h∈{1,…,H}, query ( $Q_{h}$), key ( $K_{h}$), and value ( $V_{h}$) matrices are derived from the (positionally encoded) input sequence $X_{i,1:P_{1}}=M_{i,1:P_{1}}+PE_{1:P_{1}}$ using learnable linear projections. The scaled dot-product attention is then adopted. The output of the final Transformer encoder layer is a sequence of contextualized, deeply fused multi-scale representations $({\tilde{m}}_{i,1},{\tilde{m}}_{i,2},\ldots ,{\tilde{m}}_{i,P_{1}}),\;$here each ${\tilde{m}}_{i,k}\in {\mathbb{R}}^{D_{\text{input}\_\text{tf}}}$. This representation for each canonical interval *k* now reflects not only its explicitly integrated multi-scale characteristics but also its broader temporal context within variable *i*, with inter-scale relationships dynamically weighted and refined by the self-attention process. These enriched representations ${\tilde{m}}_{i,k}$ are subsequently utilized as node features in the dynamic graph module for modeling inter-series dependencies.

### 4.4 Inter-series dependency modeling via dynamic GNN

The interactions and correlations between different time series variables are paramount for accurate medical diagnostic prediction. To model these complex and often dynamic inter-series relationships, MTFP-DG employs a dynamic graph neural network approach. This stage operates for each canonical time interval *k*, processing the contextualized, multi-scale representations ${\tilde{m}}_{i,k}\in {\mathbb{R}}^{D_{\text{input}\_\text{tf}}}$ (the output for variable *i* at interval *k*). The process comprises two main steps: dynamic graph construction and subsequent graph neural network aggregation.

#### 4.4.1 Dynamic graph construction

For each canonical time interval $k\in \{1,\ldots ,P_{1}\}$, a unique adaptive graph $G_{k}=(V,A_{k})$ is constructed. The set of nodes V={1,…,N} represents the *N* time series variables. The core of this step is the generation of a dynamic adjacency matrix $A_{k}\in {\mathbb{R}}^{N\times N}$ that quantitatively describes the inter-variable relationships specific to this time interval *k*. The construction of $A_{k}$ leverages both the contextualized temporal embeddings ${\tilde{m}}_{i,k}$ and dynamic Fourier coefficients representing the frequency characteristics of each variable within that interval. For each variable *i* and canonical time interval *k*, dynamic Fourier coefficients $f_{i,k}\in {\mathbb{R}}^{D_{\text{fourier}}}$ are derived from the raw observations within the patch(es) corresponding to this interval *k*, using the Lomb-Scargle periodogram algorithm to handle irregularly sampled data. These Fourier coefficients are then concatenated with the contextualized temporal embeddings ${\tilde{m}}_{i,k}$ (Equation 15):

(15) $$c_{i,k}=\text{Concat}({\tilde{m}}_{i,k},f_{i,k})$$

The resulting combined feature vector $c_{i,k}\in {\mathbb{R}}^{D_{\text{comb}}}$, where $D_{\text{comb}}=D_{\text{input}\_\text{tf}}+D_{\text{fourier}}$, encapsulates both temporal context and frequency domain signatures for variable *i* at interval *k*. To learn the adaptive graph structure, these combined features for all *N* variables at interval *k*, stacked into a matrix $C_{k}\in {\mathbb{R}}^{N\times D_{\text{comb}}}$, are projected into two distinct embedding spaces to derive source and target node representations for graph learning. The source and target embeddings are computed as (Equations 16, 17):

(16) $$E_{k}^{\text{source}}=C_{k}W_{g}^{\text{source}}$$

(17) $$E_{k}^{\text{target}}=C_{k}W_{g}^{\text{target}}$$

where $W_{g}^{\text{source}},W_{g}^{\text{target}}\in {\mathbb{R}}^{D_{\text{comb}}\times D_{g}}$ are learnable weight matrices, and $D_{g}$ is the dimensionality of these graph-specific node embeddings. The dynamic adjacency matrix $A_{k}$ is then generated by applying a ReLU activation followed by a row-wise Softmax function: (Equation 18)

(18) $$A_{k}=\text{Softmax}(\text{ReLU}(E_{k}^{\text{source}}(E_{k}^{\text{target}}{)}^{⊤}))$$

This process yields a unique, directed, and weighted graph structure for each time interval *k*, reflecting the evolving inter-dependencies between variables.

The graph is constructed from both contextual temporal embeddings and interval-specific frequency descriptors because inter-variable dependencies in medical IMTS are typically dynamic and state-dependent. Using only the contextual temporal representation may under-represent associations that are primarily expressed through shared rhythmic or oscillatory behavior, whereas relying only on spectral descriptors would ignore the current temporal state encoded by the Transformer output. By combining both sources of information, the learned adjacency matrix is expected to capture complementary aspects of inter-variable coupling. In addition, we infer a distinct graph for each canonical interval instead of using a single static adjacency matrix, since physiological relationships may evolve over time. The separate source and target projections allow potentially asymmetric directed relations to be represented, and the row-wise Softmax yields normalized edge weights that stabilize interval-specific graph aggregation.

#### 4.4.2 Graph neural network aggregation

With the dynamically constructed adjacency matrix $A_{k}$ for each time interval *k*, a Graph Convolutional Network (GCN) layer (Kipf and Welling, 2016) is applied to model the patch-level dynamic correlations between variables. The initial node features for the GCN at interval *k* are the contextualized, multi-scale temporal embeddings ${\tilde{m}}_{i,k}$. Let ${\tilde{M}}_{k}\in {\mathbb{R}}^{N\times D_{\text{input}\_\text{tf}}}$ be the matrix stacking these features for all *N* variables at interval *k*. The GCN layer updates the node features by aggregating information from neighboring nodes as defined by $A_{k}$ and a symmetric normalization scheme commonly used in GCNs. First, self-loops are added to the adjacency matrix: ${\hat{A}}_{k}=A_{k}+I_{N}$, where $I_{N}$ is the identity matrix. Then, the symmetrically normalized adjacency matrix is computed: ${\bar{A}}_{k}={\hat{D}}_{k}^{-1/2}{\hat{A}}_{k}{\hat{D}}_{k}^{-1/2}$, where ${\hat{D}}_{k}$ is the diagonal degree matrix of ${\hat{A}}_{k}$ (with ${\hat{D}}_{k,ii}=\sum_{j}{\hat{A}}_{k,ij}$). The GCN layer operation is then (Equation 19):

(19) $${\bar{M}}_{k}=\text{GELU}({\bar{A}}_{k}{\tilde{M}}_{k}W_{\text{gnn}})$$

where $W_{\text{gnn}}\in {\mathbb{R}}^{D_{\text{input}\_\text{tf}}\times D_{\text{gnn}\_\text{out}}}$ is the learnable weight matrix for the GCN layer, and GELU is the activation function. The output dimensionality $D_{\text{gnn}\_\text{out}}$ is set to $D_{\text{input}\_\text{tf}}$ to maintain feature dimension consistency. The GNN aggregation step is performed for each canonical time interval *k* using its specific graph structure derived from $A_{k}$, with shared GNN parameters $W_{\text{gnn}}$. The output of this stage is a sequence of further refined representations $\left({\bar{m}}_{i,1},\ldots ,{\bar{m}}_{i,P_{1}}\right)$, where each ${\bar{m}}_{i,k}\in {\mathbb{R}}^{D_{\text{input}\_\text{tf}}}$ has now incorporated information about inter-variable dependencies pertinent to its specific time interval *k*. These representations are then passed to the final prediction layer.

### 4.5 IMTS forecasting and loss functions

#### 4.5.1 Prediction layer (IFAN output)

To generate predictions for the $H^{\prime }$ future time points $\tau =\{\tau_{1},\tau_{2},\ldots ,\tau_{H^{\prime }}\}$ (where $T<\tau_{1}<\;\ldots <\tau_{H^{\prime }}\leq T+H$), a final latent representation for each variable *i*, ${\bar{m}}_{i,P_{1}}$, is utilized. This vector, representing the state derived from the last historical canonical time interval, encapsulates comprehensive multi-scale, dual-domain, intra-series, and inter-series characteristics up to time *T*. The prediction for each variable *i* at each future query timestamp $\tau_{k^{\prime }}$ is then generated using an output layer structured as a time-aware Irregular Fourier Analysis Network (IFAN) layer. This IFAN prediction layer, denoted $\phi_{\text{pred}}$, takes the context vector ${\bar{m}}_{i,P_{1}}$ and the (normalized) future timestamp $\tau_{k^{\prime }}$ as input (Equation 20):

(20) $$z_{i,k^{\prime }}=\phi_{\text{pred}}({\bar{m}}_{i,P_{1}},\tau_{k^{\prime }})$$

The internal computation of $\phi_{\text{pred}}(x_{\text{ctx}},t_{\text{fut}})$ is defined as (Equation 21):

(21) $$\phi_{pred}({\text{x}}_{ctx},t_{fut})=[\text{cos}(W_{p}^{\left(\text{pred}\right)}{\text{x}}_{ctx}+U_{p}^{\left(\text{pred}\right)}t_{fut})\;∥\;\text{sin}(W_{p}^{\left(\text{pred}\right)}{\text{x}}_{ctx}+U_{p}^{\left(\text{pred}\right)}t_{fut})\;∥\;\sigma (B_{\bar{p}}^{\left(\text{pred}\right)}+W_{\bar{p}}^{\left(\text{pred}\right)}{\text{x}}_{ctx}+V_{\bar{p}}^{\left(\text{pred}\right)}t_{fut})]$$

$W_{p}^{\left(\text{pred}\right)},U_{p}^{\left(\text{pred}\right)},W_{\bar{p}}^{\left(\text{pred}\right)},V_{\bar{p}}^{\left(\text{pred}\right)},B_{\bar{p}}^{\left(\text{pred}\right)}$ are learnable parameters specific to this prediction layer, and σ is the GELU activation function. The resulting feature vector $z_{i,k^{\prime }}$ is subsequently passed through a final projection layer to produce the forecast ${\hat{y}}_{i,k^{\prime }}\in {\mathbb{R}}^{D_{v}}$, where $D_{v}$ is the dimensionality of the observed variable (Equation 22):

(22) $${\hat{y}}_{i,k^{\prime }}=W_{\text{out}}z_{i,k^{\prime }}+b_{\text{out}}$$

with $W_{\text{out}}$ and $b_{\text{out}}$ being the learnable weights and bias of this output projection.

#### 4.5.2 Loss functions

The MTFP-DG model is trained end-to-end by minimizing a specifically designed Medical Time-Aware Loss Function ( ${ℒ}_{\text{MTA}}$). This loss function is formulated based on Mean Squared Error (MSE) and incorporates a time-aware weighting mechanism that directly reflects the actual temporal distance into the future for each prediction. The rationale for this design is to enhance the model’s predictive accuracy, particularly for longer forecast horizons which are quantitatively defined by these temporal intervals. Such emphasis is often critical in medical applications for enabling proactive interventions and effective planning. By prioritizing performance on these more challenging long-range predictions, the loss function encourages the model to learn more robust representations of underlying temporal dynamics. The Loss Function ${ℒ}_{\text{MTA}}$ is defined as (Equation 23):

(23) $${ℒ}_{\text{MTA}}=\frac{1}{N\cdot H^{\prime }}\sum_{i=1}^{N}\sum_{k^{\prime }=1}^{H^{\prime }}w(\tau_{k^{\prime }},T){\left({\hat{y}}_{i,k^{\prime }}-y_{i,k^{\prime }}\right)}^{2}$$

In this formulation, ${\hat{y}}_{i,k^{\prime }}$ represents the predicted value for variable *i* at the $k^{\prime }$-th future time point $\tau_{k^{\prime }}$, and $y_{i,k^{\prime }}$ is the corresponding ground truth value. *N* is the number of variables, and $H^{\prime }$ is the number of future time points in the prediction horizon. The temporally adaptive weight $w(\tau_{k^{\prime }},T)$ makes the loss function “time-aware” by directly incorporating the actual time interval between the end of the observation window *T*, and the future prediction time point $\tau_{k^{\prime }}$. This weight is defined as (Equation 24):

(24) $$w(\tau_{k^{\prime }},T)=1+\alpha (\tau_{k^{\prime }}-T)$$

where α is a non-negative hyperparameter that controls the sensitivity of the loss to the forecast horizon length $\left(\tau_{k^{\prime }}-T\right)$. A value of α=0 would revert to a standard MSE (averaged per point), while α>0 ensures that predictions for time points further into the future are assigned a linearly increasing weight, thus receiving greater emphasis during training. The timestamps $\tau_{k^{\prime }}$ and *T* are assumed to be on a consistent numerical scale where their difference represents a meaningful duration. The choice of this MSE-based, time-interval-weighted loss function is motivated by factors pertinent to medical forecasting. The squared error term of MSE makes the loss particularly sensitive to large deviations, which is critical in medical settings where significant prediction errors can have severe consequences. By applying weights $w(\tau_{k^{\prime }},T)$ that are directly proportional to the actual future time interval, $L_{\text{MTA}}$ steers the model to improve its performance on long-interval predictions in a manner that precisely reflects the temporal extent of the forecast. This is vital because forecasting uncertainty naturally accumulates over longer actual time durations, and a dedicated focus, scaled by this duration, helps in learning the deeper, underlying patterns necessary for such extrapolations. This emphasis on longer-term accuracy aligns with the clinical need for foresight, where the “length” of an interval is measured in actual time units, allowing for more effective planning and preemptive care based on the model’s predictions. This single objective function $L_{\text{MTA}}$ thus encapsulates the primary learning goal for the MTFP-DG model, guiding it to optimize a balance between immediate and future predictive performance based on the actual temporal distance of those future points.

## 5 Results

### 5.1 Experimental setup

#### 5.1.1 Datasets

We involve five datasets, including MIMIC-III, MIMIC-IV, PhysioNet2012, PhysioNet2019, and eICU, related to the healthcare domain in order to specifically evaluate the performance of the models in the healthcare IMTS prediction task. Therefore, we randomly divided all instances in each dataset into training, validation, and testing sets with 60%, 20%, and 20%, respectively. Each patient is treated as one instance and is assigned to exactly one subset only, thereby preventing any overlap between the training, validation, and test sets. For a given random split, all compared methods are evaluated on the same partition to ensure a fair comparison. **Table 1** is a summary of these datasets.

**Table 1:** Description of the dataset.

Description	MIMIC-III	MIMIC-IV	PhysioNet2012	PhysioNet2019	eICU
Number of variables	96	94	41	34	52
Number of patients	23,457	25,072	12,000	31,474	8,214
Average observations	1.5	2.2	10.7	1.8	2.7

MIMIC-III (Johnson et al., 2016) is a widely accessible clinical database containing electronic health records of intensive care patients. After the preprocessing provided by Biloš et al (Biloš et al., 2021), we collected IMTS from 23,457 patients from the first 48 hours after their admission to the hospital, with 96 variables in each IMTS, and we obtained a missingness rate of approximately 95.73% for the time series. MIMIC-IV continued the success of MIMIC-III Johnson et al., 2020), and after preprocessing, we collected IMTS from 25,072 patients from the first 48 hours after patient admission, each with 94 variables, and we obtained a missing rate of approximately 91.35% for the time series. PhysioNet2012 (Silva et al., 2012) contains 12,000 IMTS corresponding to different patients, and each IMTS consists of a total of 41 variables of clinical signals collected at irregular intervals during the first 48 hours after the patient’s admission to the ICU. The rate of missingness of these indicators was as high as 80%. The PhysioNet 2019 challenge dataset was provided by the 2019 Physionet Sepsis Early Prediction Challenge (Reyna et al., 2020). We selected data from the first 48 hours after a patient’s admission to the ICU and filtered it to obtain 31,474 IMTS in which each patient was monitored by 34 irregular sensors, including 8 vital signs and 26 laboratory values. We obtained a missing rate of approximately 94.9% for the time series. (Despite the fact that each patient is documented on an hourly basis, with each row representing an hour of data, the data exhibit irregularities in the feature dimensions from a technical modeling perspective.) The eICU database is a large-scale clinical database containing intensive care units (ICUs) from different regions of the United States (Pollard et al., 2018). We extracted 52 measured value variables from the eICU database for the first 48 hours after ICU admission for 8214 patients. We obtained a missingness rate of approximately 89.63% for the time series.

#### 5.1.2 Implementation details

All experiments were performed on a server equipped with 4080Ti graphics card with 24G memory and we implemented MTFP-DG in Pytorch. To ensure fair comparisons, for all models compared, we always set the hidden dimension of all datasets used to 64 and chose a batch size of 32. We employ Adam optimizer for these models’ training and apply early stopping when the validation loss doesn’t decrease over 20 epochs. To mitigate randomness, we perform each experiment using five different random seeds and give the mean and standard deviation of the results. The best performing model is selected based on the loss of the validation dataset and used for testing. For each IMTS, we use the first 24 hours as the observed data to predict the queried values in the next 24 hours. For the detailed setup of MTFP-DG, we chose the multi-scale patches’ window sizes {*s*} as {1,2,4,8,24} hours for each dataset. This scale set was selected to provide a compact but sufficiently broad coverage of temporal resolutions within the 24-hour observation and 24-hour forecasting setting. To reduce the number of generated patches, we do not overlap the patch splits and keep a sliding-step patch window equal to its size. In addition, missing values in the sequences were labeled and set to indicate as missing before training. In MTFP-DG, temporal normalization is applied only to timestamps within each patch: each timestamp is linearly rescaled to [0,1) before TTCN/IFAN encoding, which standardizes the local temporal coordinate system without altering the benchmark observation sequence itself. The dimensions of the temporal embedding $D_{t}$ and the variable embedding $D_{g}$ were set to 10, balancing model complexity and performance; too large a size could lead to a performance crash because of possible data sparsity problems when learning semantic embeddings for certain variables. The number of heads in the Transformer, the number of layers in the GNN, and the number of blocks were chosen to be 1. We set the learning rate to 0.001 for the whole model training. For baseline methods, we start from the recommended settings in the original papers or official implementations; the reported test results correspond to the best validation configuration under the same data partition.

#### 5.1.3 Evaluation metrics

Current IMTS forecasting studies primarily use mean square error (MSE) for evaluation (Equation 25). To provide a more comprehensive assessment of model performance, we also incorporate the mean absolute error (MAE) (Equation 26), which is a widely used evaluation metric in classical time series forecasting. Additionally, we calculate the mean relative error (MRE), root mean square error (RMSE), and mean absolute percentage error (MAPE). These metrics all reflect the degree of difference between the estimated values and the true values (smaller values are better).

(25) $$\text{MSE}=\frac{1}{N}\sum_{n=1}^{N}\frac{1}{Q_{n}}\sum_{j=1}^{Q_{n}}{\left({\hat{y}}_{j}^{\left(n\right)}-y_{j}^{\left(n\right)}\right)}^{2}$$

(26) $$\text{MAE}=\frac{1}{N}\sum_{n=1}^{N}\frac{1}{Q_{n}}\sum_{j=1}^{Q_{n}}❘{\hat{y}}_{j}^{\left(n\right)}-y_{j}^{\left(n\right)}❘$$

where ${\hat{y}}_{j}^{\left(n\right)}$, $y_{j}^{\left(n\right)}$ are the predicted and true values at the *j*-th prediction query time point for the *n*-th variable, respectively.

#### 5.1.4 Baselines

In order to assess the validity of MTFP-DG and establish a comprehensive measure for the underdeveloped IMTS prediction task, we included 10 relevant baselines for fair comparison, covering the SOTA models: the PatchTST (Nie et al., 2022), TimesNet (Wu et al., 2022), Neural Flows (Biloš et al., 2021), GRU ODE (De Brouwer et al., 2019), Warpformer (Zhang et al., 2023), FourierGNN (Yi et al., 2023), CRU (Schirmer et al., 2022), Latent ODEs (Rubanova et al., 2019), Medformer (Wang et al., 2024), TITD (Ji et al., 2025). We carefully select the key hyper-parameters of these models around their recommended setups. For the MTS prediction models, we input the sequences after canonical pre-alignment and include the observed times, mask information and prediction queries as additional features incorporated into these models. To make the classification baseline models suitable for the prediction task, we replace their classification output layer with the standard common practice MLP-based prediction output layer.

### 5.2 Main results

**Figure 4:**
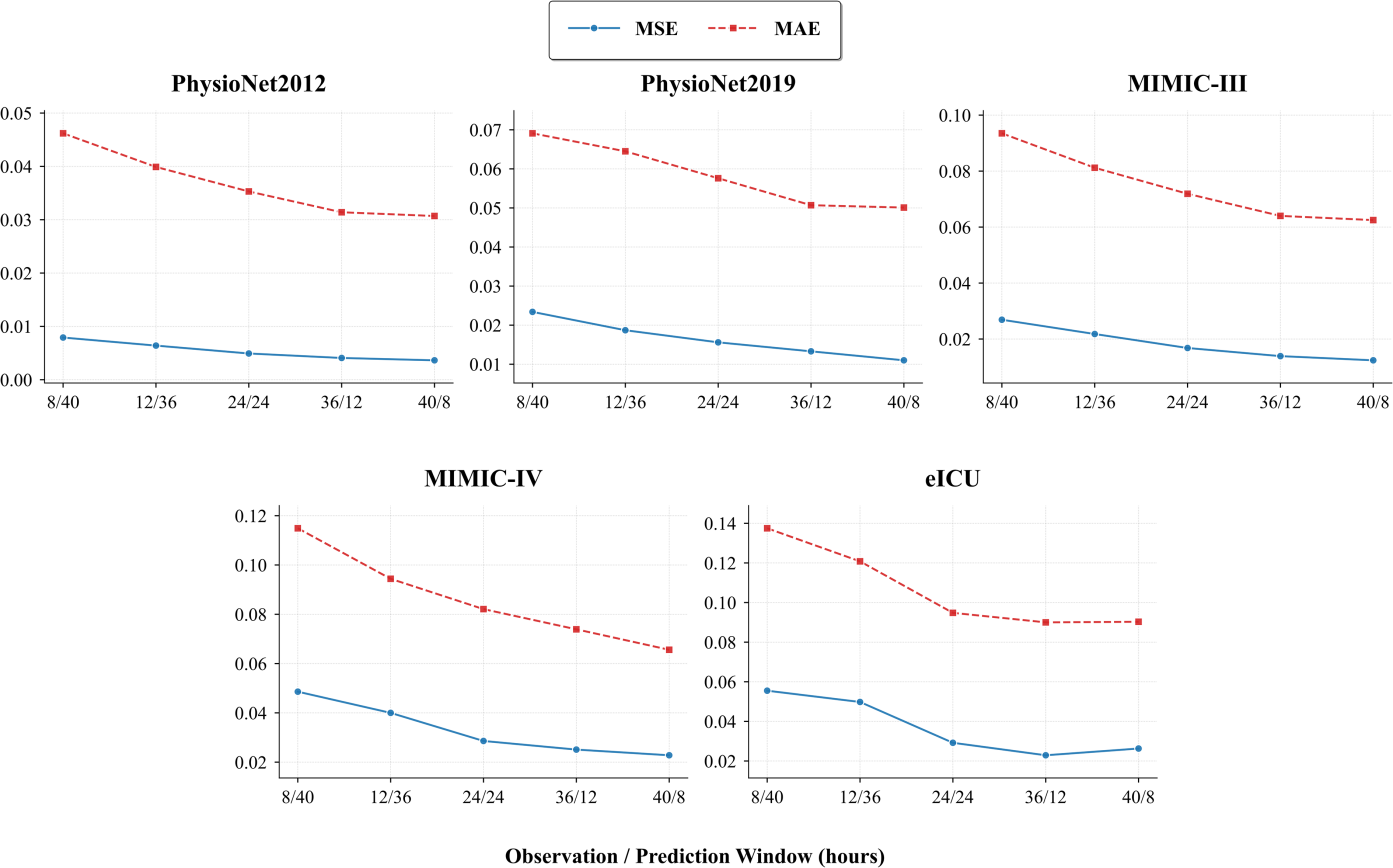
Performance of different observation and prediction window ranges.

**Table 2** reports the model prediction performance evaluated using MSE and MAE across five datasets. Overall, we observe significant performance differences among different baselines across different datasets and evaluation metrics. Some advanced methods, such as Warpformer, demonstrate outstanding performance on the PhysioNet dataset. However, despite extensive hyperparameter tuning, these methods do not exhibit comparable efficacy on datasets based on MIMIC. On the other hand, graph-based methods utilising frequency domain analysis, such as FourierGNN, perform poorly on PhysioNet. We speculate that the fundamental cause is related to the degree of alignment between the effective patterns of the specific dataset and the unique assumptions held by the specific model. It can be seen that MTFP-DG maintains the best performance across all datasets, even outperforming the second-best baseline by 10% on some datasets. Additionally, we observe that MTS prediction models, including patch-based models and GNN-based models, do not achieve consistent competitive performance in IMTS prediction. This suggests that directly applying these two techniques to IMTS is ineffective in handling the challenging internal and cross-time series modeling. Furthermore, existing IMTS prediction models have not achieved satisfactory performance, possibly because they fail to effectively simulate the correlations between time series to enhance prediction performance. Several key factors influence the performance of MTFP-DG. Its multi-scale patch mechanism, combined with dual-domain encoders (TTCN and IFAN), has been optimized to effectively capture the inherent fine-grained short-term dynamics and macro-level long-term trends in irregular multi-modal time series, while naturally addressing the asynchrony of medical data through patch-level temporal alignment. Additionally, by avoiding interpolation steps and employing a masking technique, noise introduced by these processes is mitigated, preserving the original feature distribution. We also tested the performance of these models on longer and shorter prediction windows. Another factor affecting performance is the length of the observation window for each task. Shorter observation windows may limit the model’s ability to capture complex temporal patterns and blur differences within and between variables. When applied to real-world medical predictions, the goal is to strike a balance between implementing predictions as early as possible and achieving the best possible prediction results. The results are shown in **Figure 4**.

**Table 2:** Overall performance evaluated by MSE and MAE (mean ± standard deviation). The best and second-best results are highlighted in bold and underlined, respectively. .

Algorithm	PhysioNet2012		PhysioNet2019		MIMIC-III		MIMIC-IV		eICU	
	MSE×10^–3^	MAE×10^–2^	MSE×10^–2^	MAE×10^–2^	MSE×10^–2^	MAE×10^–2^	MSE×10^–2^	MAE×10^–2^	MSE×10^–2^	MAE×10^–2^
PatchTST	12.41 ± 0.26	6.13 ± 0.17	4.26 ± 0.27	10.52 ± 0.17	3.84 ± 0.03	12.59 ± 0.11	4.25 ± 0.03	11.62 ± 0.09	5.16 ± 0.13	15.61 ± 0.15
TimesNet	16.72 ± 0.13	6.25 ± 0.05	4.85 ± 0.52	11.84 ± 0.12	5.97 ± 0.09	13.88 ± 0.08	5.26 ± 0.06	12.53 ± 0.07	6.46 ± 0.17	13.86 ± 0.21
Neural Flow	7.22 ± 0.08	4.75 ± 0.07	2.62 ± 0.08	7.27 ± 0.11	1.93 ± 0.06	8.21 ± 0.19	3.15 ± 0.09	9.59 ± 0.12	3.97 ± 0.12	10.98 ± 0.10
GRU-ODE	5.95 ± 0.62	4.31 ± 0.09	2.37 ± 0.06	6.92 ± 0.09	1.87 ± 0.11	7.74 ± 0.21	3.53 ± 0.08	9.71 ± 0.15	4.94 ± 0.15	12.46 ± 0.22
Warpformer	5.62 ± 0.28	4.22 ± 0.15	1.99 ± 0.05	6.58 ± 0.12	2.11 ± 0.05	8.59 ± 0.11	3.83 ± 0.13	10.34 ± 0.18	4.56 ± 0.18	12.84 ± 0.15
FourierGNN	8.35 ± 0.36	5.64 ± 0.12	3.28 ± 0.29	8.68 ± 0.19	1.98 ± 0.03	8.08 ± 0.08	3.16 ± 0.16	8.84 ± 0.11	4.27 ± 0.13	11.46 ± 0.08
CRU	8.59 ± 0.24	5.78 ± 0.17	2.59 ± 0.51	7.24 ± 0.26	1.97 ± 0.02	7.93 ± 0.19	3.68 ± 0.06	9.15 ± 0.13	5.14 ± 0.07	14.11 ± 0.14
Latent ODEs	6.61 ± 0.26	4.37 ± 0.12	2.45 ± 0.16	7.11 ± 0.34	1.89 ± 0.19	8.11 ± 0.46	3.34 ± 0.17	9.54 ± 0.13	4.71 ± 0.16	12.51 ± 0.13
Medformer	5.51 ± 0.15	4.15 ± 0.11	2.15 ± 0.10	6.72 ± 0.15	1.81 ± 0.09	7.85 ± 0.15	3.25 ± 0.11	9.21 ± 0.14	4.15 ± 0.10	11.55 ± 0.12
TITD	5.88 ± 0.21	4.35 ± 0.18	1.92 ± 0.08	6.45 ± 0.10	1.89 ± 0.13	7.99 ± 0.25	3.11 ± 0.08	8.75 ± 0.10	3.85 ± 0.11	11.21 ± 0.15
**MTFP-DG**	**4.92 ± 0.09**	**3.53 ± 0.05**	**1.56 ± 0.04**	**5.76 ± 0.08**	**1.68 ± 0.07**	**7.19 ± 0.09**	**2.86 ± 0.03**	**8.21 ± 0.09**	**2.92 ± 0.08**	**9.48 ± 0.07**

Bold values indicate the best performance.

### 5.3 Interpretability analysis of the learned dynamic graphs

**Figure 5:**
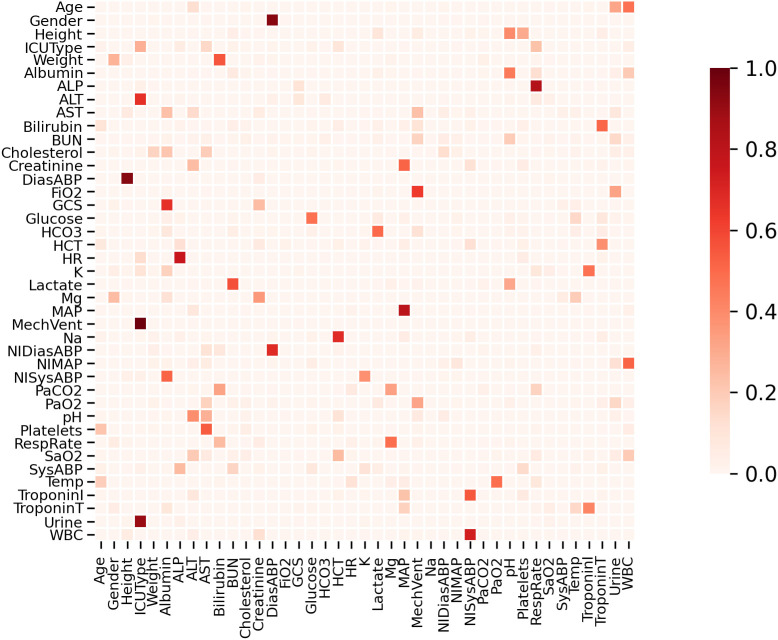
Verification of PhysioNet 2012 variable correlation. Verification of meaningful variable associations on PhysioNet2012 using the learned graph structure. The heatmap visualizes an episode-aggregated adjacency summary derived from the dynamic graphs to highlight salient inter-variable dependencies. We emphasize that these dependencies are associative (non-causal) and are used to support plausible, hypothesis-generating explanations of the forecasting behavior.

We also examined the visualization of the learned adaptive graph structure adjacency matrix to analyze how it works under different conditions. Overall, we found that the learned adjacency matrices are typically sparse, indicating that our model attempts to learn true correlations from the data rather than simply aggregating these variables. For PhysioNet2012, as shown in **Figure 5**, we observed that our model can learn insightful correlations between different patient metric variables. Additionally, through the graph structure learning process, some latent, more complex correlations can be automatically discovered from the data. For example, it indicates a high correlation between blood urea nitrogen and lactate levels. Typically, BUN levels reflect kidney function. Impaired kidney function leads to reduced clearance of urea and lactate, resulting in lactate accumulation.

To address clinical interpretability more systematically, we explicitly describe how graph-based explanations are derived from the learned time-indexed adjacency matrices. For each test episode and each canonical interval *k*, the dynamic graph module produces an adaptive adjacency matrix $A_{k}\in {\mathbb{R}}^{N\times N}$, where $A_{\text{k}}(i,j)$ represents the learned *directed* edge weight from variable *i* to variable *j* at interval *k*. To obtain an episode-level summary that is readable by clinicians, we compute a temporal aggregation (Equation 27)

(27) $$\bar{A}=\;\frac{1}{P_{1}}\sum_{k=1}^{P_{1}}A_{k},$$

and interpret $\bar{A}(i,j)$ as the average strength of the learned association from variable *i* to *j* over the observation window. For visualization and narrative interpretation, we report the most salient edges by selecting the top-ranked entries of $\bar{A}$ (after normalization to [0,1] for display), and we group them according to physiological subsystems (see below). Importantly, because $A_{k}$ is learned from observational EHR data to support forecasting, the edges should be interpreted as *associative dependencies* useful for summarizing time-varying cross-variable influence patterns, rather than as evidence of causal mechanisms.

Beyond sparsity, the learned relations align with physiologically coherent systems. (*i*) *Renal–metabolic coupling:* strong edges frequently appear among blood urea nitrogen (BUN), creatinine, urine output, and lactate, consistent with reduced renal clearance contributing to lactate retention during impaired kidney function. (*ii*) *Oxygenation–hemodynamics:* we observe connections between oxygenation markers (e.g., ${\text{SpO}}_{2}$, and when available ${\text{PaO}}_{2}$/ ${\text{FiO}}_{2}$) and mean arterial pressure (MAP) or heart rate (HR), reflecting the interplay between tissue perfusion and oxygen delivery. (*iii*) *Electrolyte/acid–base balance:* associations among sodium, chloride, bicarbonate (and anion gap when available) emerge, consistent with acid–base regulation. These patterns are not imposed by priors: the graphs are learned per interval from contextualized temporal states and irregularity-aware spectral features. We caution that the edges are *associative*, not causal; nevertheless, they provide clinically plausible hypotheses about time-varying cross-variable influence.

To quantify the reproducibility of the learned dynamic graphs, we leverage the five independently trained models (different random seeds) already used for performance evaluation (Section 5.1.2). For each trained model, we compute the episode-aggregated adjacency summary $\bar{A}$ on the test set and binarize it by retaining the top-10% of edge weights, yielding a sparse mask $B^{\left(r\right)}\in {\left\{0,1\right\}}^{N\times N}$ for run *r*. Structural consistency is assessed via two metrics averaged over all $(\begin{array}{c} 5\\ 2 \end{array})=10$ pairs of runs: (i) the pairwise Jaccard similarity of the binarized masks (Equation 28):

(28) $$J(B^{\left(r\right)},B^{\left(r^{\prime }\right)})=\frac{❘B^{\left(r\right)}\cap B^{\left(r^{\prime }\right)}❘}{❘B^{\left(r\right)}\cup B^{\left(r^{\prime }\right)}❘},$$

and (ii) the Pearson correlation of the vectorized continuous edge weight matrices $\text{vec}({\bar{A}}^{\left(r\right)})$ and $\text{vec}({\bar{A}}^{\left(r^{\prime }\right)})$. The results are reported in **Table 3**. Across all five datasets, the average pairwise Jaccard similarity of the binarized top-10% adjacency masks ranges from 0.78 to 0.83, and the average Pearson correlation of the full edge weight distributions ranges from 0.85 to 0.91, indicating that the dominant graph topology is largely preserved across independent training runs. The moderate variance is consistent with the stochasticity inherent in deep neural network training.

**Table 3:** Stability of learned graph structures across five independent training runs, measured by the average pairwise Jaccard similarity of binarized top-10% adjacency masks and the average pairwise Pearson correlation of continuous edge weight vectors (mean ± std over 10 run pairs).

Dataset	Jaccard similarity	Pearson correlation
PhysioNet 2012	0.81 ± 0.03	0.89 ± 0.04
PhysioNet 2019	0.83 ± 0.04	0.91 ± 0.03
MIMIC-III	0.79 ± 0.04	0.86 ± 0.05
MIMIC-IV	0.80 ± 0.05	0.88 ± 0.04
eICU	0.78 ± 0.05	0.85 ± 0.06

To examine whether similar physiological association patterns emerge across datasets, we restrict the episode-aggregated adjacency $\bar{A}$ to the 19-variable shared subset between PhysioNet 2012 and MIMIC-III (**Table 4**, Section 5.7) and compute the Pearson correlation between the resulting 19×19 edge weight matrices obtained on the respective test sets. The cross-dataset Pearson correlation is 0.72±0.06 (mean ± std over five seeds), indicating moderately high consistency across the two independent ICU cohorts. This recurrence across independently collected ICU databases suggests that these patterns reflect genuine physiological co-variation in critically ill patients rather than dataset-specific artefacts. Residual divergence is attributable to differences in patient population characteristics, measurement protocols, variable granularity, and missingness rates between the two databases. We caution that this cross-dataset comparison is confined to the shared variable subset and that the learned associations remain observational and non-causal.

**Table 4:** Shared variables between the MIMIC-III 96-variable subset and PhysioNet 2012 used in our cross-database evaluation.

PhysioNet 2012 variable	MIMIC-III variable (96-var subset)	Note
Albumin	Albumin	lab
ALP	Alkaline Phosphatase	lab
ALT	Alanine Aminotransferase	lab
AST	Aspartate Aminotransferase	lab
Bilirubin	Bilirubin, Total	lab
BUN	Urea Nitrogen	synonym mapping
Glucose	Glucose	lab
HCO3	Bicarbonate	lab
HCT	Hematocrit	lab
K	Potassium	lab
Lactate	Lactate	lab
Mg	Magnesium	lab
Na	Sodium	lab
PaCO2	pCO2	blood gas; ensure consistent sampling source
PaO2	pO2	blood gas; ensure consistent sampling source
pH	pH	blood gas
Platelets	Platelet Count	lab
WBC	White Blood Cells	lab
Urine	Foley + Void + Urine Out Incontinent	aggregated output

### 5.4 Ablation study

To further investigate the impact of independent components in MTFP-DG, we evaluated the performance of MTFP-DG and several of its variants on five datasets to highlight the importance of key design choices. (1) -P: Removing patches and adopting a standardized pre-aligned representation; (2) -M: Removing multi-scale patches and replacing them with standard time series patches (Nie et al., 2022); (3) -F: Using only time-domain encoding for patch embedding, removing frequency-domain features and eliminating frequency-domain-related operations in subsequent modules; (4) -T: Removing the Transformer module from the model; (5) -G: Removing dynamic graph augmentation and not modeling inter-variable relationships. Complete denotes the model with no ablations.

**Table 5** shows the results of the model ablation. It can be seen that removing any component may lead to a performance decline compared to the complete model. From these results, we observe that removing patches causes a significant performance decline across all datasets. Medical datasets have highly irregular sampling patterns, with observation timepoints varying by patient and variable, which demonstrates that patching irregular time series indeed facilitates subsequent intra- and inter-time-series modeling in IMTS. Removing frequency domain features leads to a decline in the model’s predictive ability for periodic signals. Although these medical time series data have high missing rates, many of the variables actually contain periodicity. By comparing the results of removing dynamic graph augmentation, we find that for physiological signal prediction tasks, the intrinsic features of variables are more important than their dynamic patterns. This makes sense because there are significant semantic differences between these signals, and without effectively identifying them, it is difficult to accurately describe their interrelationships.

**Table 5:** Ablation results of MTFP-DG on five datasets.

Ablation	PhysioNet2012		PhysioNet2019		MIMIC-III		MIMIC-IV		eICU	
	MSE×10^–3^	MAE×10^–2^	MSE×10^–2^	MAE×10^–2^	MSE×10^–2^	MAE×10^–2^	MSE×10^–2^	MAE×10^–2^	MSE×10^–2^	MAE×10^–2^
-P	5.28 ± 0.08	3.95 ± 0.04	1.93 ± 0.11	7.64 ± 0.07	1.95 ± 0.07	7.97 ± 0.03	3.46 ± 0.06	9.70 ± 0.04	3.67 ± 0.02	10.97 ± 0.04
-M	5.10 ± 0.06	3.68 ± 0.05	1.71 ± 0.03	6.24 ± 0.05	1.72 ± 0.03	7.23 ± 0.02	2.88 ± 0.05	8.26 ± 0.02	3.25 ± 0.07	10.64 ± 0.09
-F	5.17 ± 0.12	3.72 ± 0.03	1.78 ± 0.04	6.30 ± 0.06	1.75 ± 0.06	7.31 ± 0.06	2.91 ± 0.06	8.31 ± 0.03	3.38 ± 0.03	10.76 ± 0.08
-T	5.19 ± 0.09	3.75 ± 0.06	1.83 ± 0.04	6.37 ± 0.06	1.77 ± 0.11	7.29 ± 0.03	2.93 ± 0.04	8.29 ± 0.04	3.38 ± 0.02	10.79 ± 0.08
-G	10.61 ± 0.16	5.43 ± 0.07	3.46 ± 0.02	10.67 ± 0.09	3.58 ± 0.03	11.08 ± 0.04	4.06 ± 0.08	11.59 ± 0.04	4.77 ± 0.06	12.49 ± 0.11
Complete	**4.92 ± 0.09**	**3.53 ± 0.05**	**1.66 ± 0.04**	**6.06 ± 0.08**	**1.68 ± 0.07**	**7.19 ± 0.09**	**2.86 ± 0.03**	**8.21 ± 0.09**	**2.92 ± 0.08**	**9.48 ± 0.07**

Bold values indicate the best performance.

### 5.5 Effect of multi-scale patches

**Figure 6:**
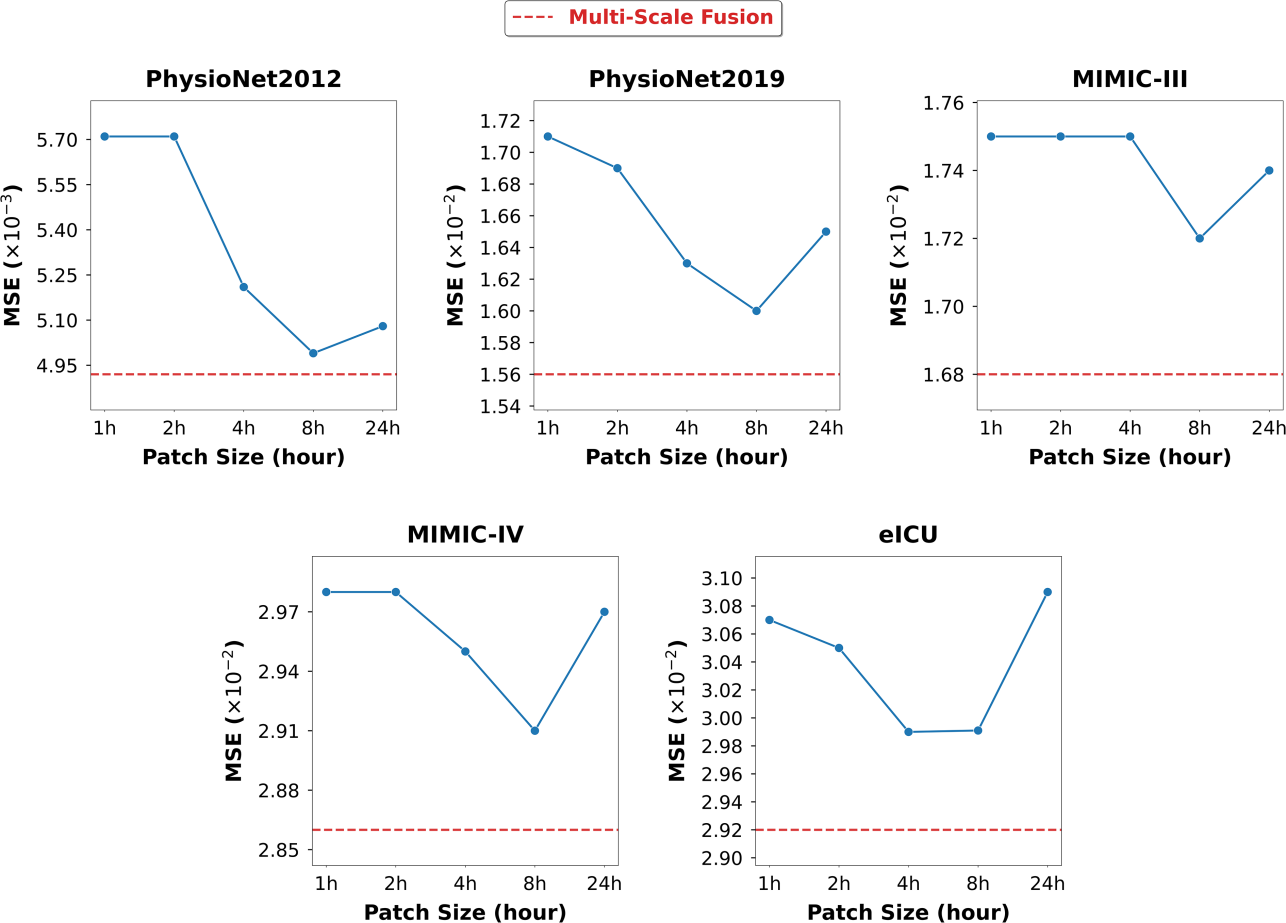
Effects of different patch sizes and multi-scale patches.

On medical datasets containing both short-term and long-term patterns, a single resolution patch size may prevent the model from adequately capturing short-term fluctuations, leading to degraded prediction performance. This underscores the importance of multi-scale patch encoding. **Figure 6** illustrates the impact of different patch window sizes and multi-scale patches on various datasets. We can observe that the effect of patch size on performance varies across different datasets. Specifically, for PhysioNet and MIMIC, performance peaks when the patch size reaches 8 hours. This may be attributed to the sparse nature of many physiological signals, where time spans shorter than four hours may not contain sufficient observations to effectively capture local patterns within sub-series. However, as the patch size increases beyond this point, we observe a decline in model performance. When selecting overly large data patch sizes, while more long-term patterns may be captured, sensitivity to short-term, fine-grained changes is lost, thereby adversely affecting detailed analysis within and between time series. From another perspective, the optimal patch size can be selected by comprehensively considering the sizes of prediction and observation windows. Long-term predictions and observations typically involve larger patch sizes to better capture trend semantics within patches and long-term dependencies across time series. The multi-scale patches used in this study can effectively reduce this issue, as they allow for the simultaneous capture of features across different time scales.

### 5.6 Sensitivity and efficiency analysis

**Figure 7:**
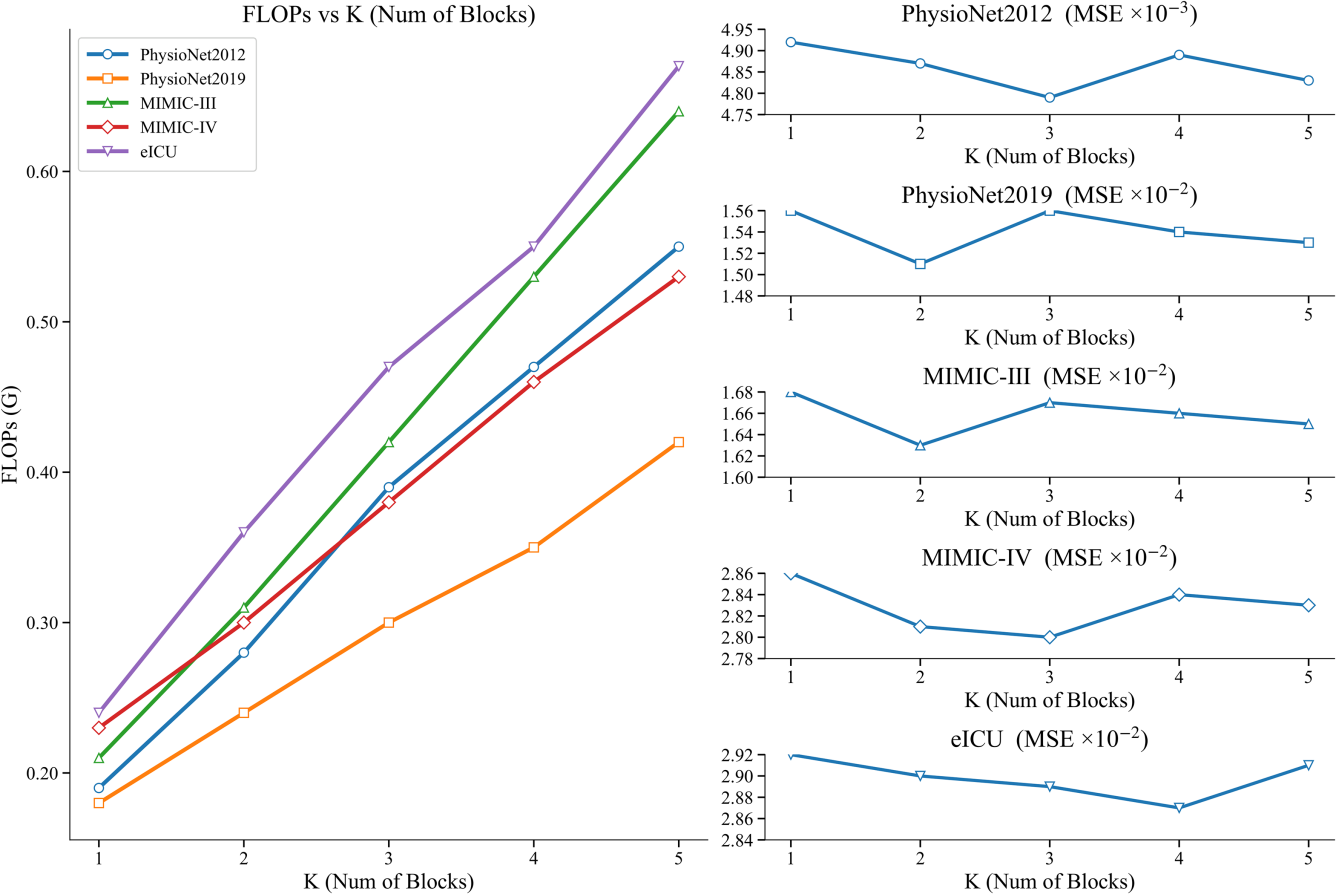
Effect of different block K.

To further assess the robustness of MTFP-DG, we conducted a parameter sensitivity analysis with respect to the number of intra- and inter-time series modeling blocks *K*, as shown in **Figure 7**. The computational cost exhibits an approximately linear increase with larger *K*. We observe that stacking multiple *K* blocks appears to hold potential for achieving better performance. However, it also incurs more expensive computational overhead. Therefore, we select *K* = 1 as the primary experiment.

Medical irregular multivariate time series (IMTS) often exhibit substantial missingness and are subject to measurement noise; moreover, missingness patterns can be informative in real-world electronic health records. Therefore, beyond hyper-parameter sensitivity, we systematically evaluate the robustness of the proposed method under controlled data-quality degradations.

We evaluate robustness by perturbing the *test set* only while keeping the trained model parameters fixed, which mimics practical deployment where data quality may deteriorate at inference time. All perturbations are applied on top of the original missingness mask. Unless otherwise stated, perturbations are performed in the standardized feature space using the training-set statistics (mean and standard deviation) to ensure consistent noise scaling across variables. For each perturbation level, we repeat the perturbation with five random seeds and report mean ± standard deviation for MSE/MAE.

Let $M\in {\left\{0,1\right\}}^{T\times D}$ denote the original observation mask, where $M_{t,d}=1$ indicates an observed value. For a target additional missingness rate ρ∈{0.1, 0.2, 0.3, 0.4, 0.5}, we create a perturbed mask $\tilde{M}$ by masking a subset of originally observed entries ( $M_{t,d}=1$) according to the following two patterns: (1) **Point-wise MCAR**: independently set ${\tilde{M}}_{t,d}=0$ with probability ρ for each entry where $M_{t,d}=1$. (2) **Block-wise missingness**: for each variable *d*, we randomly sample contiguous time blocks whose total length is approximately $\rho \cdot T_{d}^{\text{obs}}$ (where $T_{d}^{\text{obs}}$ is the number of originally observed time indices for variable *d*) and set ${\tilde{M}}_{t,d}=0$ for all indices inside the sampled blocks. This block-wise setting approximates clinically plausible dropouts caused by interrupted monitoring or delayed lab measurements. We then evaluate all models using the perturbed mask $\tilde{M}$ without re-training. The quantitative robustness results are summarized in **Table 6**.

**Table 6:** Robustness to increased missingness under two masking patterns: point-wise MCAR and blockwise missingness. Performance (mean ± std over five perturbation seeds) under additional missingness rate ρ on five datasets using MTFP-DG.

Additional missingness ρ	Masking pattern	PhysioNet 2012		PhysioNet 2019		MIMIC-III		MIMIC-IV		eICU	
		MSE×10^–3^	MAE×10^–2^	MSE×10^–2^	MAE×10^–2^	MSE×10^–2^	MAE×10^–2^	MSE×10^–2^	MAE×10^–2^	MSE×10^–2^	MAE×10^–2^
0.0 (original)	Point-wise MCAR	4.92 ± 0.09	3.53 ± 0.05	1.56 ± 0.04	5.76 ± 0.08	1.68 ± 0.07	7.19 ± 0.09	2.86 ± 0.03	8.21 ± 0.09	2.92 ± 0.08	9.48 ± 0.07
	Block-wise missingness	4.92 ± 0.09	3.53 ± 0.05	1.56 ± 0.04	5.76 ± 0.08	1.68 ± 0.07	7.19 ± 0.09	2.86 ± 0.03	8.21 ± 0.09	2.92 ± 0.08	9.48 ± 0.07
0.1	Point-wise MCAR	5.13 ± 0.14	3.80 ± 0.12	1.61 ± 0.09	5.99 ± 0.15	1.73 ± 0.10	7.39 ± 0.13	2.95 ± 0.06	8.46 ± 0.16	3.01 ± 0.11	9.76 ± 0.17
	Block-wise missingness	5.03 ± 0.13	3.74 ± 0.11	1.58 ± 0.11	5.82 ± 0.18	1.70 ± 0.19	7.26 ± 0.16	2.89 ± 0.14	8.29 ± 0.13	2.95 ± 0.15	9.57 ± 0.18
0.2	Point-wise MCAR	5.35 ± 0.15	3.89 ± 0.12	1.68 ± 0.18	6.19 ± 0.14	1.80 ± 0.13	7.72 ± 0.15	3.07 ± 0.16	8.82 ± 0.16	3.14 ± 0.16	10.18 ± 0.18
	Block-wise missingness	5.12 ± 0.21	3.78 ± 0.13	1.60 ± 0.18	5.92 ± 0.17	1.73 ± 0.16	7.39 ± 0.16	2.94 ± 0.17	8.44 ± 0.15	3.00 ± 0.16	9.75 ± 0.19
0.3	Point-wise MCAR	5.63 ± 0.21	4.01 ± 0.14	1.76 ± 0.19	6.51 ± 0.20	1.90 ± 0.18	8.13 ± 0.14	3.23 ± 0.18	9.29 ± 0.18	3.30 ± 0.15	10.72 ± 0.17
	Block-wise missingness	5.29 ± 0.13	3.87 ± 0.11	1.66 ± 0.15	6.12 ± 0.12	1.78 ± 0.15	7.64 ± 0.15	3.04 ± 0.11	8.72 ± 0.14	3.10 ± 0.14	10.07 ± 0.18
0.4	Point-wise MCAR	5.85 ± 0.29	4.12 ± 0.17	1.83 ± .025	6.77 ± 0.19	1.97 ± 0.18	8.45 ± 0.24	3.36 ± 0.18	9.65 ± 0.24	3.43 ± 0.19	11.14 ± 0.26
	Block-wise missingness	5.92 ± 0.27	4.10 ± 0.12	1.85 ± 0.26	6.85 ± 0.16	2.00 ± 0.14	8.55 ± 0.18	3.40 ± 0.25	9.76 ± 0.18	3.47 ± 0.23	11.27 ± 0.21
0.5	Point-wise MCAR	6.33 ± 0.42	4.28 ± 0.19	1.98 ± 0.31	7.32 ± 0.33	2.14 ± 0.31	9.14 ± 0.29	3.64 ± 0.28	10.43 ± 0.31	3.71 ± 0.36	12.05 ± 0.37
	Block-wise missingness	7.12 ± 0.41	4.62 ± 0.12	2.17 ± 0.29	8.00 ± 0.15	2.34 ± 0.25	10.00 ± 0.21	3.98 ± 0.34	11.41 ± 0.19	4.06 ± 0.36	13.18 ± 0.29

To further study the benefits of MTFP-DG’s patching and modeling approach on the model’s efficiency, we present the efficiency of MTFP-DG against baselines on PhysioNet 2012 in **Table 7**. MTFP-DG yields a favorable complexity–latency profile. It is markedly lighter than patch-based Transformers (PatchTST, Warpformer) and Medformer, and only slightly heavier than the most compact graph/RNN baselines (FourierGNN, CRU, GRU-ODE), while avoiding the continuous-time integration overheads of Neural Flow and Latent ODE. Overall, MTFP-DG offers a strong accuracy–efficiency trade-off.

**Table 7:** Efficiency comparison between MTFP-DG and baseline models on PhysioNet 2012 in terms of parameter count (M), FLOPs (G), training time per epoch (s), and inference time per sample (ms).

Model	Params (M)	FLOPs (G)	Train (s/epoch)	Infer (ms/sample)
PatchTST	0.61	0.52	39.84	2.47
TimesNet	0.45	0.31	30.26	2.04
Neural Flow	0.53	0.43	50.18	3.06
GRU-ODE	0.11	0.11	25.36	1.46
Warpformer	0.52	0.63	45.72	3.08
FourierGNN	0.21	0.21	20.64	1.06
CRU	0.16	0.16	22.58	1.18
Latent ODE	0.31	0.41	60.32	4.96
Medformer	0.64	0.57	41.82	2.62
TITD	0.45	0.36	31.44	2.12
MTFP-DG	0.26	0.21	20.36	1.82

Beyond relative efficiency, practical edge deployment depends on whether the inference latency fits the clinical refresh cycle. In our studied ICU forecasting setting, patient records are updated periodically, hence a “real-time” constraint refers to producing an updated forecast within the data-acquisition/update interval. Under this scenario, model computation is unlikely to be the bottleneck for bedside/edge integration.

### 5.7 Cross-database generalization across ICU cohorts

All five datasets used in this study are derived from ICU cohorts (MIMIC-III/IV, PhysioNet 2012 and 2019, and eICU). To provide a stricter assessment of generalization under realistic distribution shifts, we conduct cross-database evaluation: we train the model on a source ICU database and directly evaluate it on a different target database. Cross-dataset generalization can be evaluated; however, for the evaluation to be valid and reliable, several necessary prerequisites must be satisfied. In particular, the prediction targets must be unified, and the input space should be restricted to the core physiological variables shared by both datasets. After preliminary experiments, we chose to conduct cross-dataset evaluation between MIMIC-III and PhysioNet 2012. These experiments are important for demonstrating the robustness of the proposed model across different institutions.

Cross-database evaluation between MIMIC-III and PhysioNet 2012 requires aligning the input variable set, because the two databases record partially different clinical measurements. Following our MIMIC-III preprocessing (a 96-variable subset) and the official PhysioNet 2012 variable definitions, we identify an explicit shared set of variables and restrict cross-database experiments to this shared set to ensure semantic consistency and reproducibility. Specifically, the shared set contains 18 laboratory/blood-gas variables with a one-to-one mapping (e.g., *BUN* in PhysioNet 2012 corresponds to *Urea Nitrogen* in MIMIC-III), plus an aggregated urine-output variable constructed by summing urine-related outputs in MIMIC-III (**Table 4**). When variables have different naming conventions, we report the exact mapping; when a PhysioNet variable represents a specific clinical concept (e.g., arterial blood gases), we only include the corresponding measurements in MIMIC-III when the sampling source is consistent; otherwise, we exclude that variable from the shared set to avoid a mismatched clinical definition. **Table 8** presents the results of cross-dataset evaluation.

**Table 8:** Cross-database generalization using MTFP-DG. The model is trained on the source database and evaluated on the target database.

Train → Test	MSE×10^–2^	MAE×10^–2^
MIMIC-III → PhysioNet 2012	1.03 ± 0.11	5.21 ± 0.12
PhysioNet 2012 → MIMIC-III	3.45 ± 0.18	10.20 ± 0.21

## 6 Discussion

To gauge face validity, two board-certified intensivists independently reviewed N=30 randomly sampled test episodes (balanced across datasets) and rated each forecast explanation as *clinically plausible* vs. *not plausible* using a 3-point rubric subsequently binarized for agreement analysis. Overall agreement was 80.0% ( 24/30 cases), with Cohen’s κ=0.57 (percentile bootstrap 95% CI ≈[0.37, 0.75]), indicating moderate inter-rater reliability. Consensus “plausible” was reached in 53.3% ( 16/30) of episodes, and at least one rater marked “plausible” in 73.3% ( 22/30). Typical justifications included renal–metabolic coupling (rising BUN/creatinine with falling urine output preceding lactate increases) and oxygenation–hemodynamics links $({\text{SpO}}_{2}$with MAP/HR during hypotension). Disagreements concentrated in episodes with sparse or asynchronous measurements, discordant chart notes, or rapidly shifting states where explanations aggregated limited evidence. This assessment is retrospective, pilot-scale, and not powered for hypothesis testing; it involves two raters from a single institution, uses a binarized plausibility rubric (potentially compressing nuance), and does not establish causal validity or prospective clinical impact. Accordingly, we interpret these results as preliminary face validity warranting a larger, preregistered study with task-specific endpoints and multi-center participation.

In this context, a “forecast explanation” refers to a structured summary derived from the learned dynamic graphs and the corresponding forecasts. Concretely, for each reviewed episode we summarized (i) the predicted trajectories over the target horizon, and (ii) a graph-derived edge set obtained from the temporally aggregated adjacency $\bar{A}$ (top-ranked edges and their involved variables), which was then mapped to physiologically interpretable subsystems (e.g., renal–metabolic, oxygenation–hemodynamics, and electrolyte/acid–base). The clinicians were asked to judge whether these graph-derived associations and their temporal context were clinically plausible for the episode, using the stated 3-point rubric. We reiterate that this evaluation is a retrospective, pilot-scale face-validity check and does not establish causality or prospective utility.

In this paper, “clinical decision support” specifically refers to trend-oriented physiological forecasting: given a patient’s historical irregular multivariate measurements, the model predicts future trajectories of physiological variables to assist clinicians in anticipating near-future trends and prioritizing attention. We emphasize that the proposed model is evaluated as a continuous forecasting approach in this study; therefore, the primary quantitative evaluation focuses on forecasting errors. The present evidence should be interpreted with appropriate caution. First, all quantitative results are obtained on retrospective datasets. Second, our evaluation focuses on technical prediction performance and an exploratory face-validity assessment. For these reasons, we regard the main contribution of this study as methodological: MTFP-DG provides an effective framework for modeling irregular physiological time series with multi-scale, temporal-frequency, and dynamic inter-variable structure. The applicability of MTFP-DG warrants detailed consideration. **Supplementary Appendix A1** provides a comprehensive discussion of application scenarios, workflow integration strategies, and the pathway toward validation. Potential clinical translation should proceed in a staged manner. A reasonable next step is broader external validation across institutions and patient groups, followed by prospective silent deployment in the intended clinical workflow to assess calibration, robustness, latency, alert burden, and usability under real-time data acquisition.

An important limitation of this study is that all experimental datasets were derived from ICU environments, characterized by extreme irregularity and acute care dynamics. While these datasets provide a rigorous testbed for evaluating IMTS forecasting methods under challenging conditions, the generalizability of MTFP-DG to non-ICU medical scenarios, such as chronic disease management in outpatient settings, remains unvalidated. Although our multi-scale patching mechanism is designed to be adaptable across varying temporal resolutions, empirical validation on chronic disease datasets is necessary to confirm cross-domain generalizability. Future work will prioritize evaluating MTFP-DG on outpatient monitoring data to assess its applicability beyond acute care settings and explore domain adaptation techniques to enhance performance across diverse clinical contexts.

## 7 Conclusion

This paper introduces MTFP-DG, a novel deep learning framework that should be viewed primarily as a methodological advance for retrospective forecasting of medical irregular multivariate time series. By integrating a multi-scale patching mechanism, dual-domain patch embedding, a Temporal Transformer, and a dynamic Graph Neural Network, our model effectively captures complex intra- and inter-series dependencies without requiring distortion-inducing pre-alignment. Extensive experiments on five real-world medical datasets confirm that MTFP-DG is a promising framework for irregular physiological forecasting. Ablation studies further validate the critical contributions of our proposed components, particularly the multi-scale patching and dynamic graph structures, in handling the challenges of clinical time series data. Despite its strong performance, several avenues for improvement remain. The current heuristic for scale selection could be enhanced with an adaptive mechanism. The most critical future direction is to enhance clinical utility and interpretability. Our focus will be on developing methods to visualize and explain the learned dynamic relationships and, most importantly, shifting the prediction paradigm from fixed intervals to clinically-defined critical time points, such as sepsis onset or post-operative complication windows. This will more directly align the model’s predictive power with actionable clinical decision support. However, establishing clinical usefulness will require additional prospective, workflow-aware, and outcome-oriented evaluation before any real-world deployment claims can be made.

## Data Availability

The original contributions presented in the study are included in the article/**Supplementary Material**. Further inquiries can be directed to the corresponding author.
