# Supplementary material for: Medical irregular multivariate time series forecasting based on multi-scale temporal-frequency domain patch fusion and dynamic graph
Source: Front Physiol. 2026 Apr 15;17:1767037. doi: 10.3389/fphys.2026.1767037 (PMC13124508; doi:10.3389/fphys.2026.1767037)
Supplement: Supplementary file 1 [file Supplementaryfile1.pdf]

Appendix: Applications and Validation Pathway

Xueping Liu [1], **Tianyi Gong** [1], **Youru Li** [2], **Na Li** [3] **and Silu Ding** [4,*]

[1] *College of Artificial Intelligence, Shenyang Aerospace University, Shenyang, China*
[2] *College of Computer Science, Beijing University of Technology, Beijing, China*
[3] *Department of Medical Imaging, Liaoning Cancer Institute and Hospital, Shenyang, China*
[4] *Department of Radiation Oncology, The First Hospital of China Medical University, Shenyang, China*

Correspondence*:
Silu Ding
slding@cmu.edu.cn

A APPLICATIONS AND VALIDATION PATHWAY

The following scenarios are intended to illustrate possible future applications of continuous physiological forecasting and should not be interpreted as validated clinical deployment claims. In the present study, MTFP-DG is evaluated retrospectively as a forecasting model; therefore, the examples below are hypothesis-generating translational scenarios that outline how the framework might be integrated into clinical workflows after prospective validation.

Our primary evaluation focused on regression metrics (MSE, MAE) appropriate for continuous time series forecasting. However, clinical utility often depends on the ability to detect threshold-crossing events—for example, identifying when a patient's mean arterial pressure (MAP) will drop below the critical threshold of 65 mmHg, triggering a need for intervention. To assess MTFP-DG's performance in such clinically relevant binary classification tasks, we conducted a supplementary analysis.

Threshold Definition: We defined thresholds based on established guidelines and clinical practice:

- **Hypotension:** MAP $<$ 65 mmHg (associated with inadequate organ perfusion)
- **Hyperlactatemia:** Lactate $>$ 2 mmol/L (indicator of tissue hypoxia/sepsis)
- **Hypoxemia:** $SpO_2 < 90\%$ (critical oxygen desaturation)
- **Oliguria:** Urine output $<$ 0.5 mL/kg/h (acute kidney injury criterion)

For each patient in our test sets, we determined whether these thresholds were crossed within prediction horizons of 6, 12, and 24 hours. We then converted MTFP-DG's continuous predictions into binary classifications (threshold will be crossed: yes/no) and computed standard performance metrics.

Metrics: We calculated sensitivity (true positive rate), specificity (true negative rate), positive predictive value (PPV), negative predictive value (NPV), and area under the receiver operating characteristic curve (AUROC) for each variable–threshold–horizon combination. Confidence intervals (95%) were computed via bootstrapping with 1,000 resamples.

Table A.1 presents the classification performance across key variables and datasets. Several important patterns emerge:

Table A.1. Decision support performance: Sensitivity, specificity, PPV, NPV, and AUROC for predicting threshold-crossing events at different forecast horizons. Values are mean (95% CI).

Dataset	Variable	Horizon	Sens.	Spec.	PPV	NPV	AUROC
PhysioNet2019	Lactate > 2 mmol/L	6h	0.87 (0.84–0.90)	0.90 (0.88–0.92)	0.73 (0.70–0.76)	0.96 (0.94–0.97)	0.91 (0.89–0.93)
		12h	0.82 (0.79–0.85)	0.88 (0.86–0.90)	0.68 (0.65–0.71)	0.94 (0.92–0.95)	0.89 (0.87–0.91)
		24h	0.76 (0.73–0.79)	0.84 (0.82–0.86)	0.61 (0.58–0.64)	0.91 (0.89–0.93)	0.82 (0.80–0.84)
MIMIC-III	MAP < 65 mmHg	6h	0.83 (0.80–0.86)	0.87 (0.85–0.89)	0.71 (0.68–0.74)	0.93 (0.91–0.95)	0.85 (0.83–0.87)
		12h	0.78 (0.75–0.81)	0.84 (0.82–0.86)	0.66 (0.63–0.69)	0.91 (0.89–0.93)	0.81 (0.79–0.83)
		24h	0.72 (0.69–0.75)	0.80 (0.78–0.82)	0.59 (0.56–0.62)	0.88 (0.86–0.90)	0.78 (0.76–0.80)
MIMIC-IV	SpO_2 < 90%	6h	0.85 (0.82–0.88)	0.89 (0.87–0.91)	0.68 (0.65–0.71)	0.95 (0.93–0.96)	0.87 (0.85–0.89)
		12h	0.80 (0.77–0.83)	0.86 (0.84–0.88)	0.63 (0.60–0.66)	0.93 (0.91–0.94)	0.84 (0.82–0.86)
		24h	0.74 (0.71–0.77)	0.82 (0.80–0.84)	0.57 (0.54–0.60)	0.90 (0.88–0.92)	0.80 (0.78–0.82)
eICU	Urine Output < 0.5 mL/kg/h	6h	0.81 (0.78–0.84)	0.86 (0.84–0.88)	0.58 (0.55–0.61)	0.94 (0.92–0.96)	0.84 (0.82–0.86)
		12h	0.76 (0.73–0.79)	0.83 (0.81–0.85)	0.53 (0.50–0.56)	0.92 (0.90–0.94)	0.81 (0.79–0.83)
		24h	0.70 (0.67–0.73)	0.79 (0.77–0.81)	0.48 (0.45–0.51)	0.89 (0.87–0.91)	0.77 (0.75–0.79)

Key Observations:

1. **High Specificity:** Across all variables and horizons, MTFP-DG maintains specificity between 0.79 and 0.90.
2. **Horizon-Dependent Performance:** As expected, performance degrades with longer prediction horizons. At the 6-hour horizon, AUROC ranges from 0.84 to 0.91; at the 24-hour horizon, AUROC drops to 0.77–0.82. This reflects increasing uncertainty in longer-term forecasts but still maintains useful discrimination.
3. **Variable-Dependent Patterns:** Lactate prediction shows the strongest performance (AUROC 0.89–0.91 at 12 h), likely because lactate trends reflect systemic metabolic processes captured by multiple correlated variables in the MTFP-DG dynamic graphs. Urine output prediction shows relatively lower PPV (0.48–0.58), reflecting the challenge of predicting this highly variable physiological parameter.
4. **Actionability:** At the 12-hour horizon—a timeframe that provides sufficient lead time for clinical intervention while maintaining good predictive performance—MTFP-DG achieves AUROC > 0.80 for all key variables. This suggests practical utility for early warning systems.

These results demonstrate that MTFP-DG's continuous predictions can be effectively thresholded to generate binary alerts suitable for decision support systems.

However, we emphasize several critical limitations:

- **Retrospective Metrics:** These metrics represent technical performance on historical data, not validated outcomes. High AUROC does not guarantee that model-based interventions will improve patient outcomes.
- **Threshold Simplification:** Clinical decision-making involves complex consideration of patient context, comorbidities, and treatment goals—factors not captured by simple threshold-crossing events.

We now describe concrete scenarios where MTFP-DG could provide actionable decision support and outline strategies for workflow integration.

Context: Sepsis is a leading cause of ICU mortality, where early recognition and intervention (e.g., antibiotics within 1 hour and source control) are critical. Current screening tools (qSOFA and SOFA scores) are threshold-based and may lag behind the onset of pathophysiologic deterioration.

MTFP-DG Solution: Using the PhysioNet2019 sepsis dataset, our experiments demonstrate that MTFP-DG effectively predicts trends in sepsis-associated biomarkers (lactate, white blood cell count, temperature, and procalcitonin) 12–24 hours in advance. As shown in Table A.1, lactate elevation prediction achieves AUROC 0.89 at the 12-hour horizon.

Workflow:

1. **Integrated Screening:** MTFP-DG operates alongside existing sepsis screening protocols. When a patient triggers qSOFA criteria (e.g., altered mental status or hypotension), the model provides probabilistic forecasts of key sepsis indicators over the next 24 hours.
2. **Risk Stratification:** Predictions are displayed as a sepsis risk dashboard showing:
   - Predicted lactate trend (current: 1.5 mmol/L $\rightarrow$ 12 h forecast: 2.8 mmol/L)
   - Predicted WBC trend and temperature trajectory
   - Dynamic graph highlighting correlations (e.g., lactate rising with BUN, suggesting concurrent renal hypoperfusion)
3. **Outcome:** Earlier, more targeted sepsis interventions could reduce progression to septic shock and associated organ dysfunction.

Mechanistic Insight: The dynamic graphs learned by MTFP-DG (Figure 5 in the main text) reveal physiologically coherent correlations. For instance, rising lactate coupled with declining MAP and urine output suggests distributive shock; rising lactate with elevated WBC and fever suggests a septic source. These patterns can guide differential diagnosis beyond simple threshold alerts.

We emphasize that the gap between retrospective technical validation and clinical deployment is substantial.
